# Proton Pump Inhibitors Promote Oral-to-Gut Enrichment of *S. anginosus* and Impair Anastomotic Healing via Succinic Acid-SUCNR1-NF-κB Signaling Axis

**DOI:** 10.3390/ijms27219988

**Published:** 2026-11-07

**Authors:** Xiaolong Li, Yuhao Jiang, Kai Zhang, Feng Yang, Liping Yan, Yuanliang Hu, Yiming Sun, Yuling Zhang, Guangsheng Du, Kun Yu, Xiancai Rao, Weidong Xiao

**Affiliations:** 1 The Second Clinical Medical College, Army Medical University, Chongqing 400037, China; 2 Department of Microbiology, Army Medical University, Chongqing 400037, China

**Keywords:** proton pump inhibitors, colorectal anastomosis, oral-to-gut transmission, *Streptococcus anginosus*, succinic acid, SUCNR1

## Abstract

Anastomotic leakage (AL) remains a devastating complication after colorectal cancer resection. Proton pump inhibitors (PPIs) are widely overused perioperatively, but their impact on anastomotic healing is unknown. We investigated whether and how PPIs affect colorectal anastomotic healing. Mice underwent colorectal anastomosis after oral or intraperitoneal esomeprazole. Healing was assessed by scoring, hydroxyproline, fibronectin, Masson staining, and bacterial dissemination to the spleen. Oral or gut microbiota were depleted with antibiotics, and FMT, 16S rRNA sequencing, metabolomics, and transcriptomics were performed. Both routes of PPI impaired healing and increased splenic bacterial load, which was abrogated by depletion of oral or gut microbiota. PPI enriched oral *Streptococcus anginosus* (*S. anginosus*) in the gut. FMT from PPI-treated donors recapitulated healing impairment, whereas FMT from donors with prior oral microbiota depletion did not. Succinic acid was identified as a key secreted metabolite of *S. anginosus*; the healing defect was recapitulated by succinic acid alone and attenuated by pharmacological SUCNR1 antagonism. Mechanistically, succinic acid activates SUCNR1-NF-κB signaling, upregulating pro-inflammatory cytokines (TNF-α, IL-1β, IL-6) and MMP activities. In an exploratory clinical association analysis, high-dose PPI users exhibited elevated faecal *S. anginosus* and succinic acid, which correlated with serum TNF-α and IL-6. In conclusion, PPI-induced acid suppression promotes oral-to-gut enrichment of *S. anginosus* and increases succinic acid, activating SUCNR1-NF-κB signaling to impair anastomotic healing. These findings caution against PPI overuse and suggest the succinic acid-SUCNR1-NF-κB axis as a potential therapeutic target.

## 1. Introduction

Anastomotic leakage (AL) is one of the most devastating postoperative complications following curative resection with primary anastomosis for colorectal cancer (CRC), with a reported incidence of 5–20% and an associated mortality rate of approximately 5% [1]. Survivors face an increased risk of long-term oncologic recurrence and a reduced quality of life spanning physical, mental, and financial well-being [1,2]. Even with ongoing advances in surgical techniques and perioperative care, the incidence of AL has not substantially declined, highlighting the urgent need to identify modifiable risk factors and develop effective preventive strategies.

Emerging evidence identifies the gut microbiota as a critical and modifiable determinant of anastomotic healing, with preclinical studies demonstrating that specific bacterial taxa can promote or impair collagen deposition, modulate local inflammation, and affect tissue repair [3,4,5,6]. Alongside established risk factors such as mechanical tension, intraoperative blood loss, systemic conditions (obesity, malnutrition, fluid and electrolyte disorders), and tumor-related factors [7,8,9], targeting the gut microbiota holds substantial preventive potential to reduce the incidence of anastomotic leakage [3,4,5,8,9].

Proton pump inhibitors (PPIs), prescribed to nearly one-quarter of adults worldwide [10], are among the most frequently used medications for stress ulcer prophylaxis. Appropriate indications include guideline-based stress ulcer prophylaxis and other documented conditions (e.g., gastroesophageal reflux disease, peptic ulcer disease); however, inappropriate use is widespread. Over 50% of perioperative PPI prescriptions are considered inappropriate [11], and in surgical departments specifically, this rate has been reported to be as high as 64.11% [12]. Moreover, recent clinical data have linked post-diagnosis PPI use to increased all-cause mortality in CRC patients, with higher-magnitude associations observed among surgical inpatients [13,14]. Given that impaired anastomotic healing and subsequent anastomotic leakage are significant contributors to these devastating outcomes, we hypothesized that PPIs may compromise colorectal anastomotic integrity.

The primary pharmacological action of PPIs is the potent and prolonged suppression of gastric acid secretion, achieved through covalent binding to inhibit the gastric H^+^/K^+^-ATPase of the parietal cell, thereby raising gastric pH [15,16]. Furthermore, gastric acid serves as a first line of defense against microorganisms introduced through food and saliva [17]. Therefore, when this barrier is compromised by PPI-induced hypochlorhydria, viable orally ingested bacteria can survive gastric transit and reach the lower gastrointestinal tract—a phenomenon termed oral-to-gut microbial transmission, which refers to the passage of viable oral microorganisms through the gastric barrier and their potential colonization of the lower gastrointestinal tract, shifting the gut microbial composition towards the profiles of the oral cavity [18]. Moreover, PPI-induced changes in the microbiome have been increasingly reported to be associated with various diseases, including *Clostridium difficile* infections, liver cirrhosis, inflammatory bowel disease, and colorectal cancer [19,20,21]. However, whether such drug-driven oral-to-gut transmission affects postsurgical recovery, particularly anastomotic healing, remains unknown.

In this study, we provide evidence consistent with PPI-induced gastric acid suppression promoting oral-to-gut enrichment of *S. anginosus*, which in turn impairs anastomotic healing. Furthermore, we identified succinic acid as a key metabolite of *S. anginosus*, which activates the SUCNR1-NF-κB signaling, a pathway that triggers a pro-inflammatory cytokine cascade and ultimately impairs anastomotic healing. These findings provide a mechanistic rationale for targeted prevention of this complication.

## 2. Results

### 2.1. PPI Administration Impairs Colorectal Anastomotic Healing in Mice

**Figure 1:**
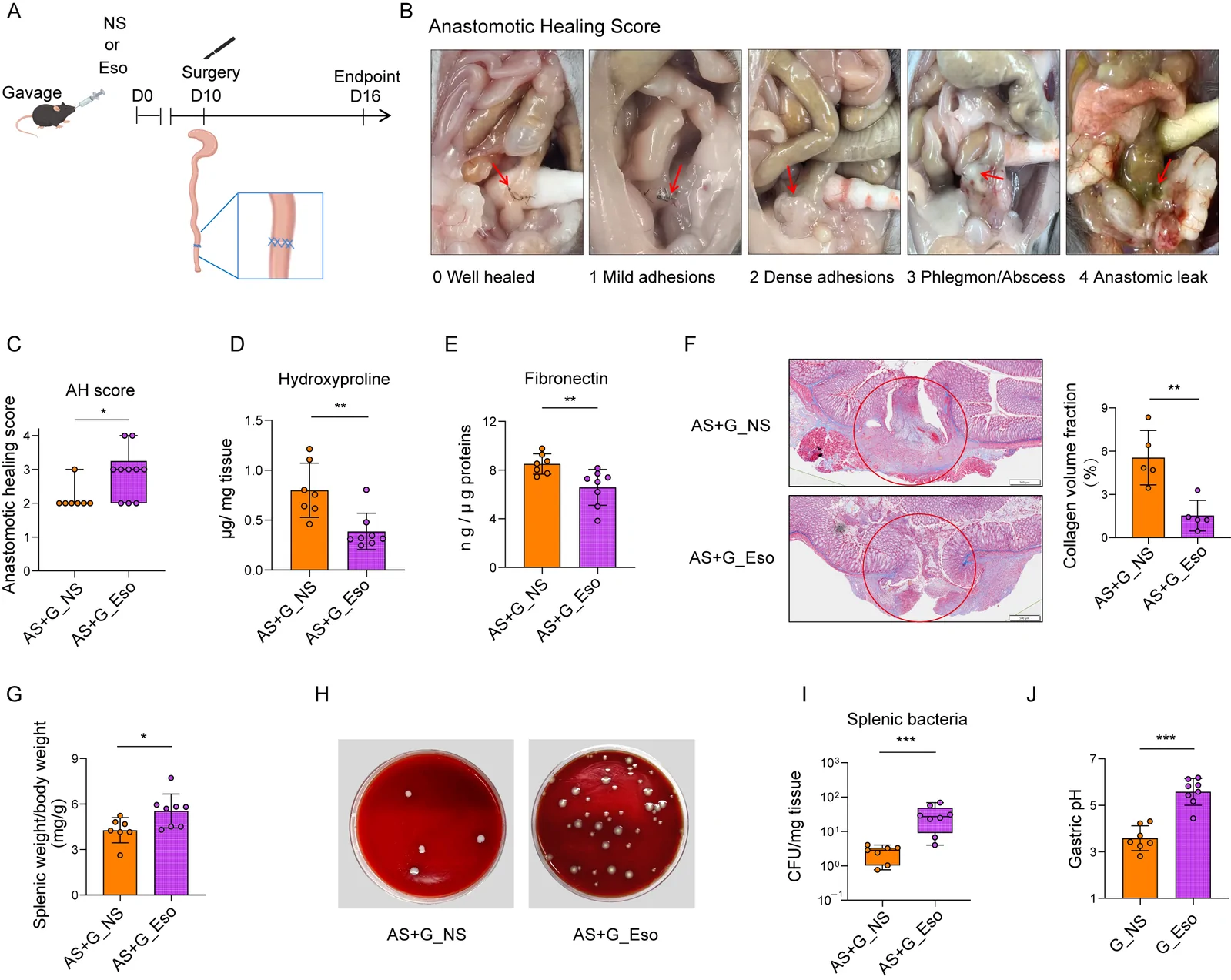
Oral PPI administration impairs colorectal anastomotic healing in mice. (**A**) Schematic of the experimental protocol for proton pump inhibitor (PPI) administration via oral gavage: mice received daily oral gavage of normal saline (NS) or Esomeprazole (Eso) for 10 consecutive days prior to surgery. Tissues were harvested for subsequent analysis on postoperative day 6. (**B**) Macroscopic score for colorectal anastomotic healing assessment in mice, with representative photographs provided for each score (0–4). The red arrows point to the anastomotic location. The scoring criteria are defined as follows: Score 0, well-healed anastomosis without adhesion; Score 1, mild filmy adhesions that were easily separable; Score 2, dense adhesions requiring sharp dissection; Score 3, phlegmon/abscess formation with localized inflammatory mass; Score 4, anastomotic leak with gross defect, intestinal content leakage and peritonitis. (**C**–**I**) Healing parameters at the anastomotic site: (**C**) anastomotic healing score (AHS, *n* = 7–10), (**D**) hydroxyproline content and (**E**) fibronectin levels (*n* = 7–8). (**F**) Representative Masson’s trichrome staining of anastomotic tissues (blue: collagen, red circle: anastomosis site; scale bars: 500 μm) and corresponding collagen volume fraction quantification (*n* = 5). (**G**) Quantification of the splenic weight-to-body weight ratio (mg/g) in mice. (**H**) Representative image of bacterial colony formation from splenic homogenates. (**I**) Quantitative bacterial load in the spleen (*n* = 7–8). (**J**) Gastric pH (*n* = 7–8). Data were presented as mean ± SD for bar chart (**D**–**G**,**J**), or as box-and-whisker plots (min to max, with median line) for AHS (**C**) and splenic bacterial load (**I**). Statistical significance was determined by two-tailed Student’s *t*-test (* *p* < 0.05, ** *p* < 0.01, *** *p* < 0.001; ns, not significant). Statistical significance for continuous variables (**D**–**G**,**J**) was determined by two-tailed Student’s *t*-test, whereas the anastomotic healing score (**C**) and splenic bacterial load (**I**) were analyzed using the Mann–Whitney U test (* *p* < 0.05, ** *p* < 0.01, *** *p* < 0.001).

To determine the effect of PPIs on anastomotic healing, we established a murine model of colorectal anastomosis and administered PPI via oral gavage for 10 days prior to surgery and continued until sacrifice on postoperative day 6 (Figure 1A). Anastomotic healing was scored based on a scoring system (Figure 1B). Oral gavage with PPI worsened anastomotic healing scores (Figure 1C). Consistently, hydroxyproline and fibronectin content, biochemical markers of extracellular matrix component deposition [4], were markedly reduced in the anastomotic tissue of PPI-gavaged mice (Figure 1D,E). Masson’s trichrome staining revealed decreased collagen volume fraction in the PPI group (Figure 1F). Furthermore, PPI-gavaged mice exhibited increased spleen-to-body weight ratio (Figure 1G) and enhanced bacterial dissemination to the spleen, evidenced by higher bacterial loads in splenic homogenates (Figure 1H,I). Gastric pH was elevated, confirming effective gastric acid suppression (3.58 ± 0.54 vs. 5.58 ± 0.57, *p* < 0.0001; Figure 1J).

Importantly, intraperitoneal (i.p.) injection of PPI, which bypasses direct exposure to the gastrointestinal lumen, similarly elevated gastric pH (3.66 ± 0.49 vs. 5.94 ± 0.68, *p* < 0.0001; Figure S1H) and produced comparable deleterious effects on anastomotic healing (Figure S1A–G). This consistent phenotypic outcome supports the interpretation that the observed healing impairment is associated with gastric acid suppression, rather than requiring local gastrointestinal luminal exposure to PPI per se.

### 2.2. Oral Microbiota Contributes to PPI-Induced Healing Impairment

**Figure 2:**
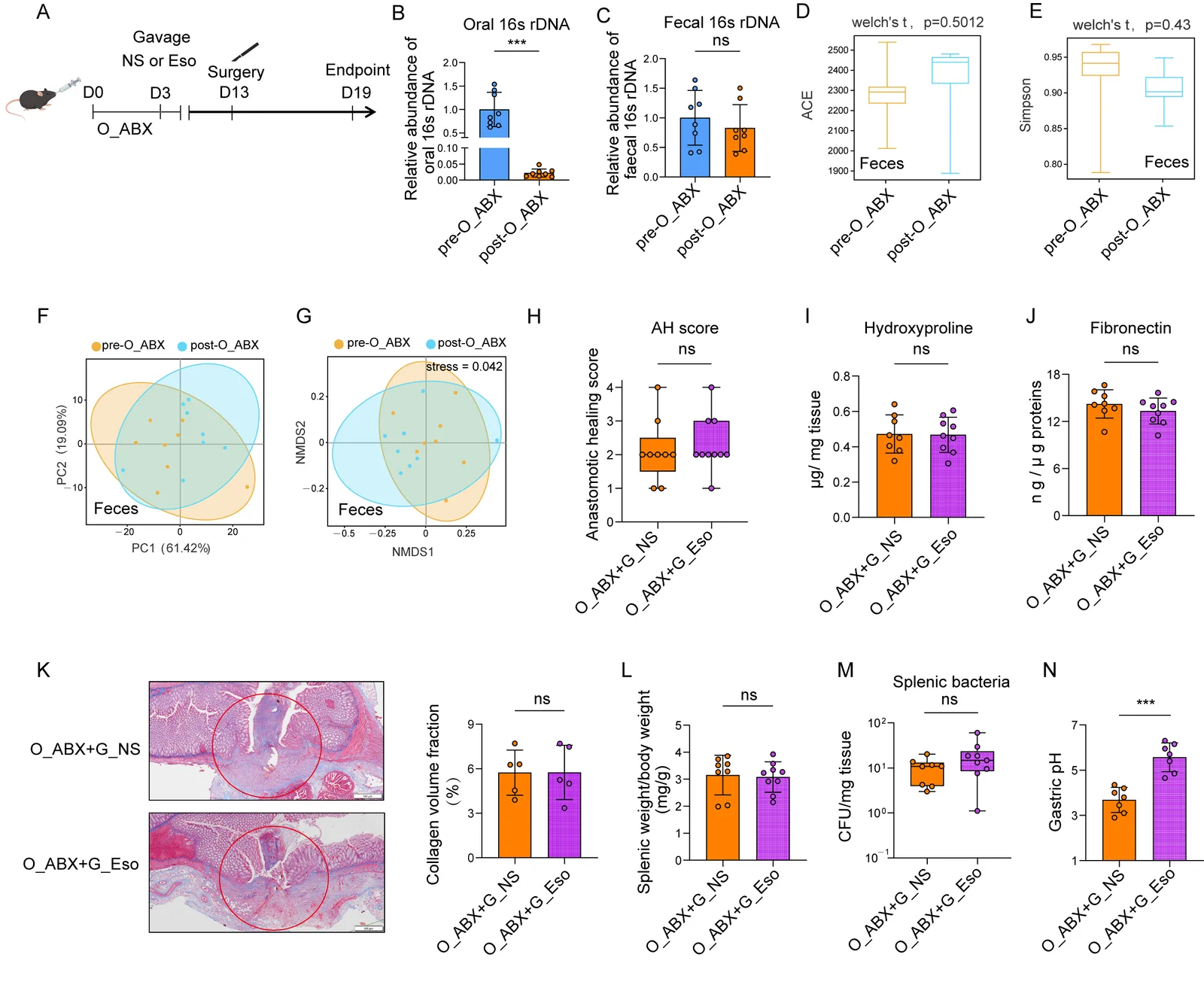
Oral microbiota depletion abrogates PPI-induced impairment of anastomotic healing. (**A**) Schematic of the experimental protocol for oral gavage of PPI administration with ABX oral swabbing: mice were given 3 consecutive days of oral swabbing with ABX, followed by 10-day daily oral gavage of NS or PPI before surgery. Tissues were harvested for subsequent analysis on postoperative day 6. (**B**) Relative abundance of oral 16S rDNA in mice before and after oral ABX swabbing (pre-O_ABX vs. post-O_ABX). (**C**) Relative abundance of faecal 16S rDNA in mice before and after oral ABX swabbing (pre-O_ABX vs. post-O_ABX). (**D**,**E**) Box plots of faecal microbiota alpha diversity indices between the pre-O_ABX and post-O_ABX groups, including ACE index (**D**) and Simpson index (**E**). (**F**) Principal component analysis (PCA) plot of faecal microbiota community structure between the pre-O_ABX and post-O_ABX groups. (**G**) Non-metric multidimensional scaling (NMDS) plot based on Bray–Curtis dissimilarity of faecal microbiota between the two groups. (**H**–**M**) Healing parameters at the anastomotic site: (**H**) anastomotic healing score (AHS, *n* = 9–10), (**I**) hydroxyproline content and (**J**) fibronectin levels (*n* = 8–9). (**K**) Representative Masson’s trichrome staining of anastomotic tissues (blue: collagen, red circle: anastomosis site; scale bars: 500 μm) and corresponding collagen volume fraction quantification (*n* = 5). (**L**) Quantification of the splenic weight-to-body weight ratio (mg/g). (**M**) Quantitative bacterial load in the spleen (*n* = 8–9). (**N**) Gastric pH (*n* = 7). Data were presented as mean ± SD for bar chart data (**B**,**C**,**I**–**L**,**N**), or as box-and-whisker plots (min to max, with median line) for AHS (**H**) and splenic bacterial load (**M**). Statistical significance for continuous variables (**B**,**C**,**I**–**L**,**N**) was determined by two-tailed Student’s *t*-test, whereas the anastomotic healing score (**H**), and splenic bacterial load (**M**) were analyzed using the Mann–Whitney U test (*** *p* < 0.001; ns, not significant).

Given that PPIs promote oral-to-gut microbial transmission [22], we next investigated whether the oral microbiota is involved. Mice received oral swabbing with a broad-spectrum antibiotic cocktail (ABX; ampicillin 20 mg/mL, vancomycin 10 mg/mL, neomycin 20 mg/mL, and metronidazole 20 mg/mL in 0.5% sodium carboxymethylcellulose) for 3 days to deplete oral bacteria, followed by gavage with PPI and anastomosis (Figure 2A). The efficacy of oral swabbing with ABX was confirmed by qPCR, which revealed a substantial reduction in total bacterial abundance from oral swabs (Figure 2B). Importantly, oral swabbing with ABX did not decrease faecal total bacterial abundance (Figure 2C). Moreover, 16S rRNA sequencing of faecal samples collected before and after this treatment revealed no significant changes in α-diversity (ACE and Simpson index; Figure 2D,E) or β-diversity (PCA, NMDS; Figure 2F,G). Remarkably, oral swabbing with ABX pretreatment largely rescued anastomotic healing in PPI-gavaged mice, as assessed by healing scores (Figure 2H), hydroxyproline (Figure 2I), fibronectin (Figure 2J), collagen deposition (Figure 2K), spleen-to-body weight ratio (Figure 2L), and bacterial dissemination to the spleen (Figure 2M).

Oral swabbing with ABX pretreatment also prevented the detrimental effects of i.p. PPI (Figure S1I–O), confirming that oral bacteria are necessary for PPI-induced healing impairment regardless of the administration route. Notably, despite elevated gastric pH after oral bacterial depletion (3.69 ± 0.55 vs. 5.57 ± 0.64, *p* < 0.0001, Figure 2N; 3.43 ± 0.46 vs. 5.32 ± 0.56, *p* < 0.0001, Figure S1P), healing impairment did not occur, indicating that acid suppression alone is insufficient and that oral microbiota are essential.

### 2.3. PPI-Induced Healing Impairment Is Abrogated by Gut Microbiota Depletion

**Figure 3:**
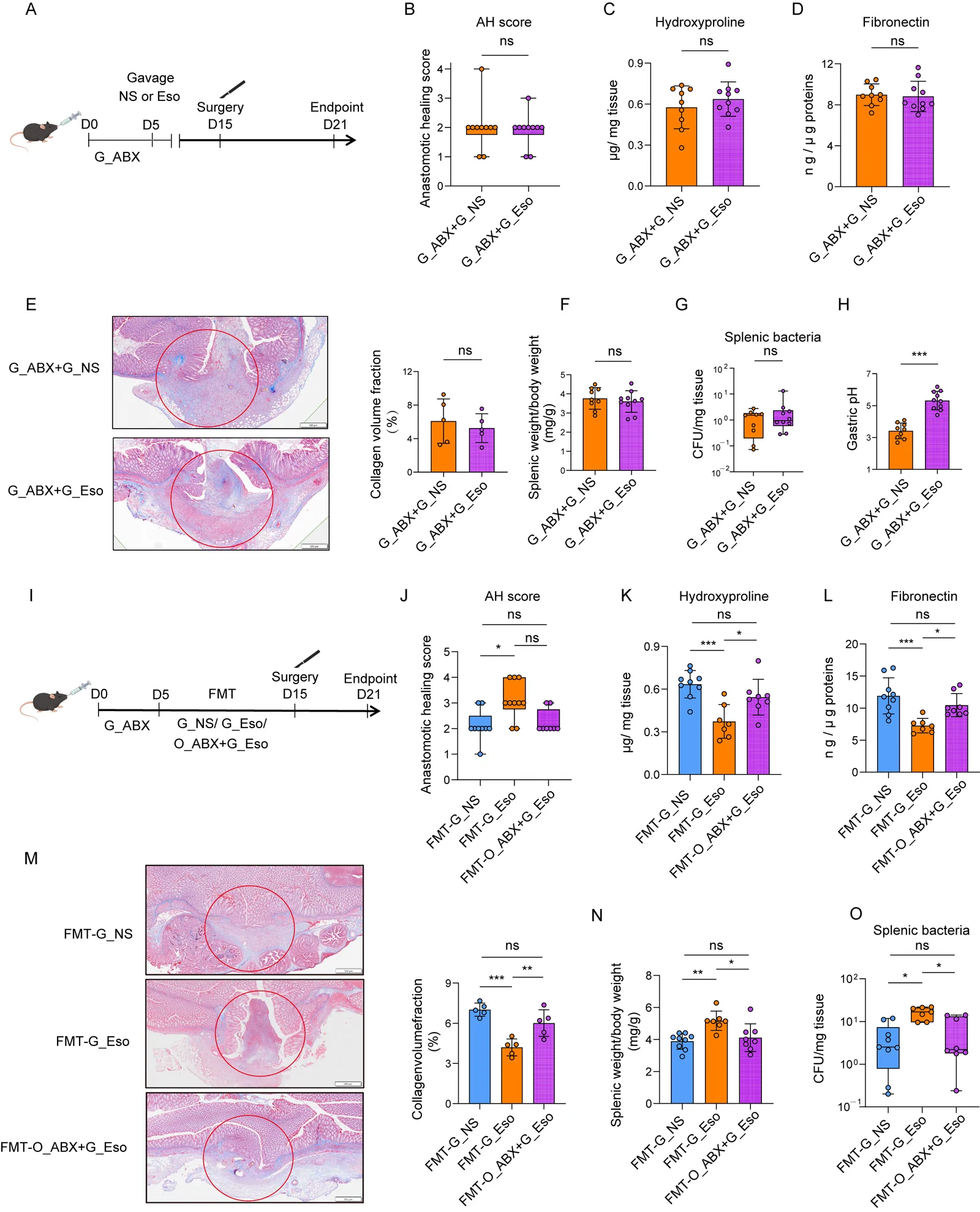
PPI-induced healing impairment is abrogated by gut microbiota depletion and is transmissible via FMT, which is associated with oral-to-gut enrichment of microbiota. (**A**) Schematic of the experimental protocol for oral gavage of PPI administration with antibiotic cocktail (ABX) gavage: mice were given 5 consecutive days of ABX gavage, followed by 10-day daily oral gavage of NS or PPI before surgery. Tissues were harvested for subsequent analysis on postoperative day 6. (**B**–**G**) Healing parameters at the anastomotic site: (**B**) anastomotic healing score (AHS, *n* = 10), (**C**) hydroxyproline content and (**D**) fibronectin levels (*n* = 9–10). (**E**) Representative Masson’s trichrome staining of anastomotic tissues (blue: collagen, red circle: anastomosis site; scale bars: 500 μm) and corresponding collagen volume fraction quantification (*n* = 5). (**F**) Quantification of the splenic weight-to-body weight ratio (mg/g). (**G**) Quantitative bacterial load in the spleen (*n* = 9–10). (**H**) Gastric pH (*n* = 9–10). (**I**) Schematic of the experimental protocol for the mouse donor FMT experiment via oral gavage: mice received 5-day pre-treatment with ABX, followed by 10 days of faecal microbiota transplantation (FMT) from donor mice treated with NS, oral gavage of Eso, or oral ABX swabbing combined with oral gavage of Eso. After the 10-day FMT intervention, mice underwent surgery, and anastomotic tissues were harvested on postoperative day 6. (**J**–**O**) Healing parameters at the anastomotic site: (**J**) anastomotic healing score (AHS, *n* = 8–10), (**K**) hydroxyproline content and (**L**) fibronectin levels (*n* = 7–9). (**M**) Representative Masson’s trichrome staining of anastomotic tissues (blue: collagen, red circle: anastomosis site; scale bars: 500 μm) and corresponding collagen volume fraction quantification (*n* = 5). (**N**) Quantification of the splenic weight-to-body weight ratio (mg/g) in mice. (**O**) Quantitative bacterial load in the spleen (*n* = 7–9). Data were presented as mean ± SD for bar chart data (**C**–**F**,**H**,**K**–**N**), or as box-and-whisker plots (min to max, with median line) for AHS (**B**,**J**) and splenic bacterial load (**G**,**O**). For two-group comparisons (**B**–**H**), statistical significance for continuous variables (**C**–**F**,**H**) was determined by a two-tailed Student’s t-test, whereas AHS (**B**) and splenic bacterial load (**G**) were analyzed using the Mann–Whitney U test. For three-group comparisons (**J**–**O**), continuous variables (**K**–**N**) were analyzed using one-way ANOVA with Tukey’s post hoc test, whereas AHS (**J**) and splenic bacterial load (**O**) were analyzed using the Kruskal–Wallis test with Dunn’s post hoc test (* *p* < 0.05, ** *p* < 0.01, *** *p* < 0.001; ns, not significant).

Then, to further test whether the gut microbiota is also required for PPI-induced healing impairment, we pre-treated mice with ABX via oral gavage for 5 days (ampicillin 200 mg/kg, vancomycin 100 mg/kg, neomycin 200 mg/kg, and metronidazole 200 mg/kg) to deplete intestinal bacteria before PPI administration and anastomosis (Figure 3A). Results showed that ABX pretreatment prevented the PPI-induced increase in anastomotic healing scores (Figure 3B), normalized hydroxyproline and fibronectin levels (Figure 3C,D), restored collagen deposition (Figure 3E), and prevented increased spleen-to-body weight ratio and bacterial dissemination to the spleen (Figure 3F,G). Notably, PPI still elevated gastric pH in ABX-gavaged mice (3.42 ± 0.49 vs. 5.31 ± 0.58, *p* < 0.0001; Figure 3H). Similarly, in i.p. PPI experiments, prior gut microbiota depletion abrogated all healing defects (Figure S2A–G), but PPI still elevated gastric pH (3.47 ± 0.46 vs. 5.19 ± 0.49, *p* < 0.0001; Figure S2H). These results indicate that gut microbiota is also necessary for PPI to impair anastomotic healing.

### 2.4. PPI-Induced Impairment of Anastomotic Healing Is Transmissible via FMT and Is Associated with Oral-to-Gut Enrichment of Microbiota

To determine whether the altered microbiota following PPI treatment is sufficient to transfer the healing impairment, we performed faecal microbiota transplantation (FMT). Donor mice were treated with oral saline, oral PPI, or oral swabbing with ABX pre-treatment followed by oral PPI. Recipient mice received the corresponding donor faeces by oral gavage and then underwent colorectal anastomosis (Figure 3I).

Recipients of PPI-donor faeces exhibited significantly worsened anastomotic healing compared with recipients of saline-donor faeces, as evidenced by increased healing scores (Figure 3J), reduced hydroxyproline and fibronectin content (Figure 3K,L) and collagen deposition (Figure 3M), elevated spleen-to-body weight ratio (Figure 3N), and higher splenic bacterial load (Figure 3O). Strikingly, recipients of faecal microbiota from mice that received oral swabbing with ABX pre-treatment (to deplete oral microbiota) prior to PPI showed no significant differences from saline-donor recipients across all healing parameters (Figure 3J–O). We obtained similar results using donors treated with intraperitoneal (i.p.) PPI. FMT from i.p. PPI treated donors recapitulated the healing defects, whereas FMT from donors that received oral bacterial depletion prior to i.p. PPI did not (Figure S2I–O).

Collectively, these results demonstrate that the PPI induced shift in gut microbiota is sufficient to transmit impaired anastomotic healing. Notably, the oral derived bacteria within this shifted microbiota—consistent with oral-to-gut transmission—may play a causal role in PPI-associated anastomotic failure.

### 2.5. PPI Treatment Is Associated with Oral-to-Gut Enrichment of Streptococcus anginosus

**Figure 4:**
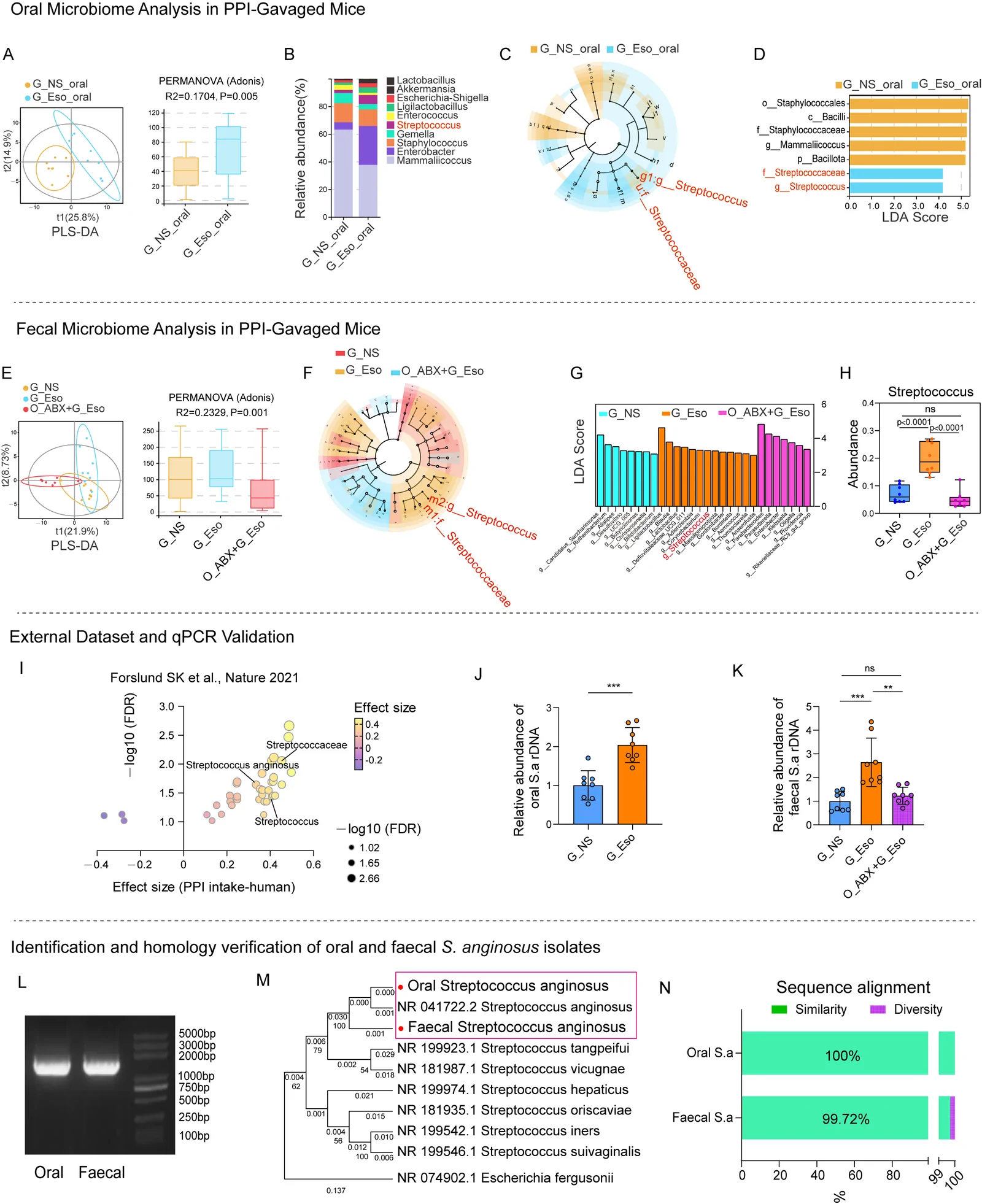
PPI treatment is associated with oral-to-gut enrichment of *Streptococcus anginosus*. (**A**) Partial least squares discriminant analysis (PLS-DA) plot demonstrating the clear separation of oral microbiota community structure between the oral gavage of normal saline (G_NS_oral group) and esomeprazole (G_Eso_oral group), with the variance interpretation rates of the t1 and t2 axes marked in parentheses (*n* = 8). The differences in overall community structure were assessed using PERMANOVA (Adonis), yielding R^2^ = 0.1704 and *p* = 0.005. (**B**) Stacked bar chart showing the relative abundance of dominant bacterial genera in oral microbiota from each experimental group. (**C**) Cladogram generated from linear discriminant analysis effect size (LEfSe) analysis. (**D**) Linear discriminant analysis (LDA) score distribution plot; the histogram shows the taxa with LDA scores greater than 4.0 (*p* < 0.05). (**E**) Partial least squares discriminant analysis (PLS-DA) plot demonstrating the distinct separation of gut microbiota community structure among the normal saline gavage (G_NS group), esomeprazole (proton pump inhibitor, PPI) gavage (G_Eso group), and oral ABX swabbing combined with PPI gavage (O_ABX+G_Eso group), with the variance interpretation rates of the t1 and t2 axes marked in parentheses (*n* = 8). PERMANOVA (Adonis) confirmed significant differences among these groups (R^2^ = 0.2329, *p* = 0.001). (**F**) Cladogram generated from linear discriminant analysis effect size (LEfSe) analysis, displaying the differentially abundant taxonomic clades among groups. (**G**) Linear discriminant analysis (LDA) scores; the histogram shows taxa with LDA scores greater than 3.0. (**H**) Box plot showing the relative abundance of *Streptococcus* in the gut microbiota of mice from each group. (**I**) Volcano plot showing differential gut microbial taxa associated with proton pump inhibitor (PPI) intake in the human cohort from Forslund SK et al. (Nature 2021) [22]. (**J**,**K**) Relative abundance of *S. anginosus* rDNA in oral swabs (**J**) and faeces (**K**) from mice receiving intragastric vehicle, PPI alone, or PPI with prior oral ABX (*n* = 8). (**L**) Agarose gel electrophoresis of 16S rRNA gene PCR amplification products from *Streptococcus anginosus* (*S.a*) isolates recovered from mouse oral cavity (Oral) and faeces (Faecal). (**M**) Neighbor-joining phylogenetic tree constructed based on 16S rRNA gene sequences, demonstrating the phylogenetic relationship between the isolated oral/faecal *S. anginosus* and reference *Streptococcus* species from GenBank. *Escherichia fergusonii* (NR 074902.1) was designated as the outgroup. Bootstrap values (1000 replicates) are shown at branch nodes, and the scale bar indicates genetic distance. (**N**) Quantitative comparison of 16S rRNA gene sequence similarity between *Streptococcus anginosus* isolated from mouse oral cavity and faeces (** *p* < 0.01, *** *p* < 0.001; ns, not significant).

To identify the bacterial taxa responsible for PPI-induced healing impairment, we performed 16S rRNA sequencing of oral swabs and faecal samples. In the oral cavity, PPI treatment induced clear beta diversity separation (PERMANOVA: R^2^ = 0.1704, *p* = 0.005; Figure 4A). Taxonomic stacking and LEfSe analysis identified *Streptococcus* as a significantly enriched taxon in the oral cavity after PPI administration (Figure 4B–D). We next analysed faecal samples from three groups: saline control, oral PPI, and oral-swab ABX pre-treatment followed by PPI gavage. Results showed significantly altered faecal beta diversity (PERMANOVA: R^2^ = 0.2329, *p* = 0.001; Figure 4E). LEfSe analysis revealed that *Streptococcus* was significantly enriched in the faeces of PPI-treated mice. (Figure 4F,G). Strikingly, the increased faecal *Streptococcus* abundance in PPI-treated mice was reversed to baseline levels by prior oral-swab ABX depletion (Figure 4H).

We next asked whether these findings translate to humans. Reanalysis of public shotgun metagenomic datasets (Forslund SK et al., Nature, 2021) [22] confirmed that human PPI users exhibit increased faecal *Streptococcus*, particularly *Streptococcus anginosus* (*S. anginosus*, Figure 4I). Then, we performed quantitative PCR targeting *S. anginosus*. Oral PPI significantly increased *S. anginosus* abundance in both the oral cavity (Figure 4J) and faeces (Figure 4K), but prior oral swabbing with ABX prevented this increase in faeces (Figure 4K). Moreover, we isolated bacterial colonies from the oral cavities and faeces of PPI-treated mice. Full-length 16S rRNA sequencing followed by phylogenetic tree analysis confirmed the isolates as *S. anginosus* (Figure 4L,M). Notably, sequence alignment revealed 99.72% homology between faecal and oral isolates from the same mouse (Figure 4N), supporting their assignment to the same species. However, this 16S rRNA-based comparison cannot establish strain-level identity or the direction of transmission.

To determine whether intraperitoneal (i.p.) PPI produces similar effects, we examined mice receiving i.p. esomeprazole. Oral swab sequencing after i.p. PPI showed clear beta diversity separation (PERMANOVA: R^2^ = 0.1834, *p* = 0.002, Figure S3A), with *Streptococcus* again enriched (Figure S3B–D). Faecal sequencing from i.p. PPI-treated mice demonstrated substantial restructuring of the gut microbiota (PERMANOVA: R^2^ = 0.3038, *p* = 0.001, Figure S3E) together with elevated *Streptococcus* abundance, which was normalised by prior oral-swab ABX (Figure S3F–H). Together, these results are consistent with PPI treatment—whether oral or intraperitoneal—inducing oral *S. anginosus* overgrowth and its subsequent enrichment in the gut. The effect is dependent on the presence of oral microbiota and is associated with gastric acid suppression, not direct intraluminal drug action.

### 2.6. S. anginosus Impairs Anastomotic Healing via Secreted Metabolites

**Figure 5:**
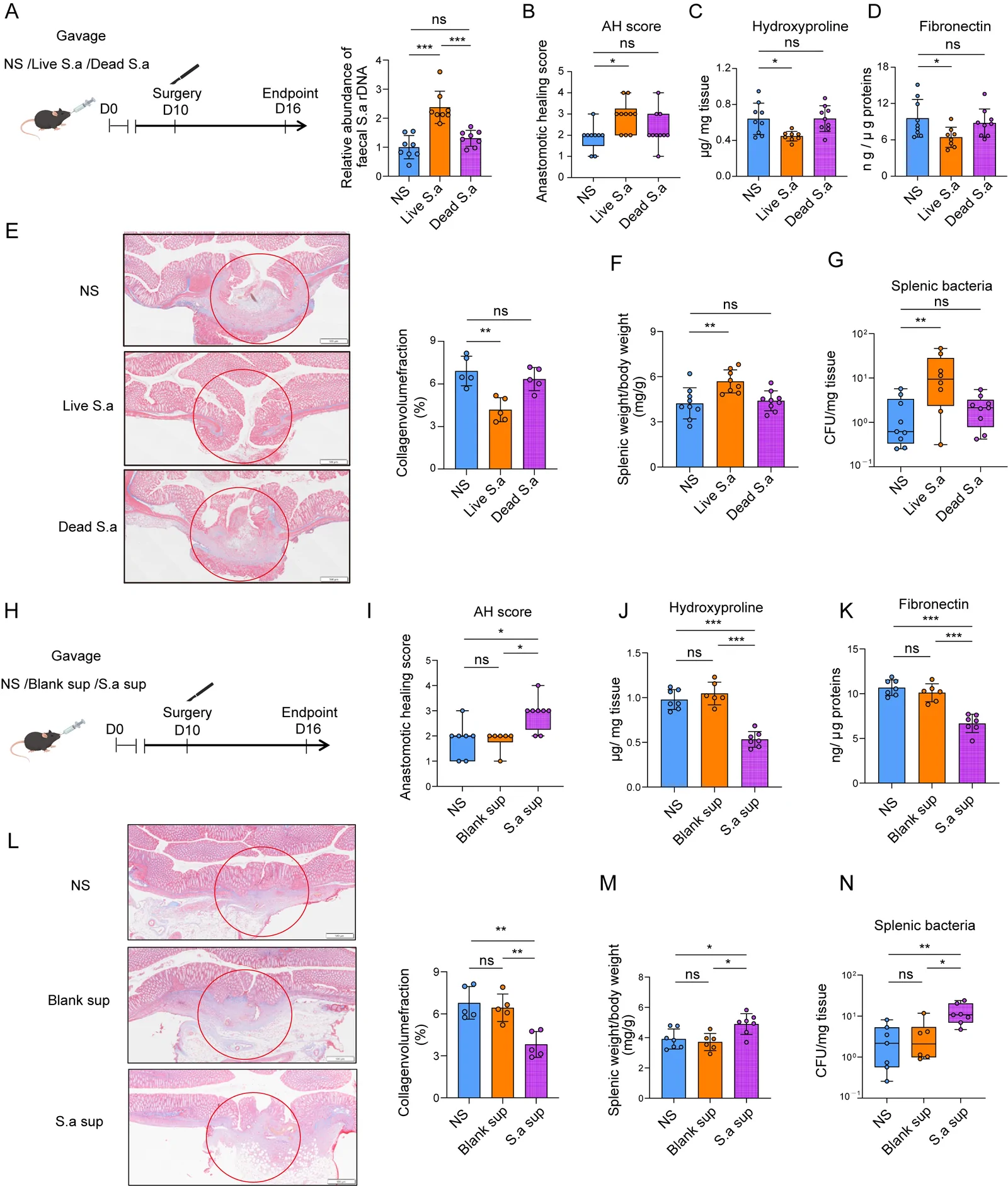
*Streptococcus anginosus* directly impairs anastomotic healing. (**A**) Schematic illustration of the experimental protocol for *Streptococcus anginosus* (*S.a*) intervention: mice were administered daily NS, live *Streptococcus anginosus* (Live *S.a*), or heat-inactivated *Streptococcus anginosus* (Dead *S.a*) for 10 days before colorectal anastomosis surgery. Tissues were collected for subsequent analysis on postoperative day 6. The right panel shows the relative abundance of faecal *S. a* rDNA measured by qPCR, verifying the successful colonization of live *S. a* (**B**–**D**) Healing parameters at the anastomotic site: (**B**) anastomotic healing score (AHS, *n* = 9–10), (**C**) hydroxyproline content and (**D**) fibronectin levels (*n* = 8–9). (**E**) Representative Masson’s trichrome staining of anastomotic tissues (blue: collagen, red circle: anastomosis site; scale bars: 500 μm) and corresponding collagen volume fraction quantification (*n* = 5). (**F**) Quantification of the splenic weight-to-body weight ratio (mg/g) in mice. (**G**) Quantitative bacterial load in the spleen (*n* = 8–9). (**H**) Schematic illustration of the experimental protocol for *S.a* culture supernatant intervention: mice were administered daily gavage of NS, blank culture supernatant (Blank sup), or *S. anginosus* culture supernatant (*S.a* sup) for 10 days before colorectal anastomosis surgery. Tissues were collected for subsequent analysis on postoperative day 6. (**I**–**K**) Healing parameters at the anastomotic site: (**I**) anastomotic healing score (AHS, *n* = 6–8), (**J**) hydroxyproline content and (**K**) fibronectin levels (*n* = 6–7). (**L**) Representative Masson’s trichrome staining of anastomotic tissues (blue: collagen, red circle: anastomosis site; scale bars: 500 μm) and corresponding collagen volume fraction quantification (*n* = 5). (**M**) Quantification of the splenic weight-to-body weight ratio (mg/g) in mice. (**N**) Quantitative bacterial load in the spleen (*n* = 6–7). Data were presented as mean ± SD for bar chart data (**C**–**F**,**J**–**M**), or as box-and-whisker plots (min to max, with median line) for AHS (**B**,**I**) and splenic bacterial load (**G**,**N**). For three-group comparisons, continuous variables (**A**,**C**–**F**,**J**–**M**) were analyzed using one-way ANOVA with Tukey’s post hoc test, whereas the anastomotic healing score (**B**,**I**) and splenic bacterial load (**G**,**N**) were analyzed using the Kruskal–Wallis test with Dunn’s post hoc test (* *p* < 0.05, ** *p* < 0.01, *** *p* < 0.001; ns, not significant).

To determine whether *S. anginosus* alone is sufficient to impair anastomotic healing and to dissect the requirement for bacterial viability, we colonized mice with live *S. anginosus* or heat-inactivated bacteria (70 °C for 30 min) prior to anastomosis and verified the successful colonization of the live bacteria (Figure 5A). Live *S. anginosus* significantly impaired anastomotic healing compared with vehicle controls, as evidenced by increased healing scores, reduced hydroxyproline and fibronectin content, decreased collagen deposition, increased spleen-to-body weight ratio, and elevated splenic bacterial load (Figure 5B–G). In contrast, heat-inactivated bacteria did not significantly affect these healing parameters (Figure 5B–G), indicating that bacterial viability is required for the deleterious effect. We next studied whether secreted bacterial products are sufficient to compromise healing. Then, mice were gavaged with supernatant from *S. anginosus* culture, with NS and blank culture medium as negative controls (Figure 5H). Notably, *S. anginosus* supernatant alone markedly worsened anastomotic healing across all parameters, whereas blank medium had no effect (Figure 5I–N), suggesting that secreted metabolites of *S. anginosus* are sufficient to impair healing.

### 2.7. Succinic Acid Impairs Anastomotic Healing Through SUCNR1

**Figure 6:**
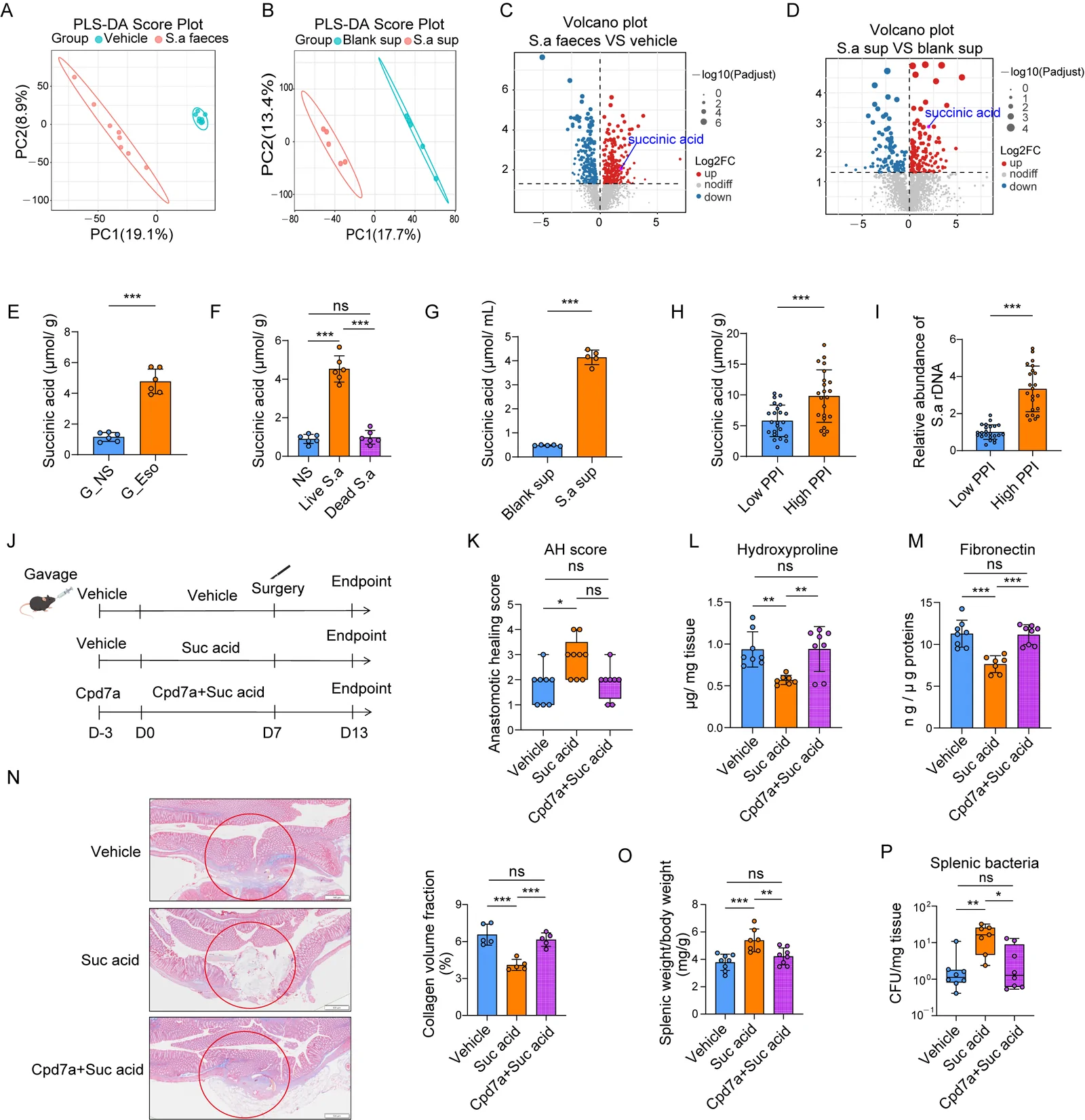
Succinic acid is a key metabolite of *S. anginosus* that impairs anastomotic healing through SUCNR1. (**A**,**B**) PLS-DA score plots of metabolomic profiles between: (**A**) faeces from mice colonized with live *S. anginosus* versus controls; (**B**) *S. anginosus* culture supernatant versus blank medium. (**C**,**D**) Volcano plot analysis of metabolites in (**C**) *S. anginosus*-colonized mouse faeces and (**D**) *S. anginosus* culture supernatant. (**E**) Faecal succinic acid levels in PPI-gavaged mice versus vehicle controls (*n* = 6). (**F**) Faecal succinic acid levels in mice colonized with live or heat-inactivated *S. anginosus* (*n* = 6). (**G**) Succinic acid levels in *S. anginosus* culture supernatant versus blank medium (*n* = 5). (**H**,**I**) Faecal succinic acid levels and *S. anginosus* abundance in patients receiving high-dose PPI (DpD ≥ 30 mg/day) versus low-dose PPI (*n* = 22 and 23, respectively). (**J**) Schematic illustration of the experimental protocol for succinic acid administration with or without SUCNR1 antagonist (Compound **7a**). (**K**–**P**) Healing parameters at the anastomotic site: (**K**) anastomotic healing score (AHS, *n* = 8–9), (**L**) hydroxyproline and (**M**) fibronectin levels (*n* = 7–8), (**N**) representative Masson’s trichrome staining of anastomotic tissues (blue: collagen, red circle: anastomosis site; scale bars: 500 μm) and corresponding collagen volume fraction quantification (n = 5), (**O**) spleen-to-body weight ratio (mg/g), and (**P**) splenic bacterial load (CFU/mg tissue, *n* = 7–8). Data are presented as mean ± SD for bar chart data (**E**–**I**,**L**–**O**), or as box-and-whisker plots (min to max, with median line) for AHS (**K**) and splenic bacterial load (**P**). For two-group comparisons, continuous variables (**E**–**I**) were analyzed using a two-tailed Student’s *t*-test. For three-group comparisons (**K**–**P**), continuous variables (**L**–**O**) were analyzed using one-way ANOVA with Tukey’s post hoc test, whereas AHS (**K**) and splenic bacterial load (**P**) were analyzed using the Kruskal–Wallis test with Dunn’s post hoc test. * *p* < 0.05, ** *p* < 0.01, *** *p* < 0.001; ns, not significant.

To identify the responsible metabolites, we performed metabolomic profiling of faeces from mice colonized with live *S. anginosus* and of *S. anginosus* culture supernatant, in parallel with their respective controls. PLS-DA score plots revealed clear separation between each experimental group and its corresponding control (Figure 6A,B). Volcano plots identified succinic acid as the only metabolite consistently and significantly upregulated in both comparisons (Figure 6C,D). Full lists of significantly increased metabolites in each comparison, including the rank of succinic acid, are provided in Table S3A,B. To further validate this finding, we examined succinic acid levels in three independent settings. Faecal succinic acid levels were significantly elevated in PPI-gavaged mice compared with vehicle controls (Figure 6E). Similarly, mice colonized with live *S. anginosus* exhibited increased faecal succinic acid levels, but heat-inactivated bacteria did not (Figure 6F). In addition, succinic acid was markedly enriched in the culture supernatant of *S. anginosus* relative to blank medium (Figure 6G). Consistent with these preclinical observations, analysis of clinical faeces from patients revealed that faecal succinic acid levels and *S. anginosus* abundance were significantly higher in those receiving high-dose PPI (DpD ≥ 30 mg/day) compared with those on low-dose (Figure 6H,I). Together, these results indicate that succinic acid is a key metabolite produced by live *S. anginosus* and is sufficient to contribute to its pathogenic effects.

Given that succinic acid signals through its cognate receptor SUCNR1, we next investigated whether succinic acid alone is sufficient to recapitulate the healing defect and, if so, whether this effect is mediated through SUCNR1. To this end, we administered succinic acid by oral gavage prior to anastomosis, with or without co-administration of a SUCNR1 antagonist (Cpd7a, Figure 6J). Succinic acid treatment alone significantly impaired anastomotic healing, as evidenced by increased healing scores, reduced hydroxyproline and fibronectin content, decreased collagen deposition, elevated spleen-to-body weight ratio, and increased splenic bacterial load (Figure 6K–P). These results indicate that succinic acid alone is sufficient to impair anastomotic healing in a manner that is pharmacologically consistent with SUCNR1 dependence.

### 2.8. Succinic Acid-SUCNR1 Signaling Activates NF-κB and Promotes Inflammation to Impair Anastomotic Healing

**Figure 7:**
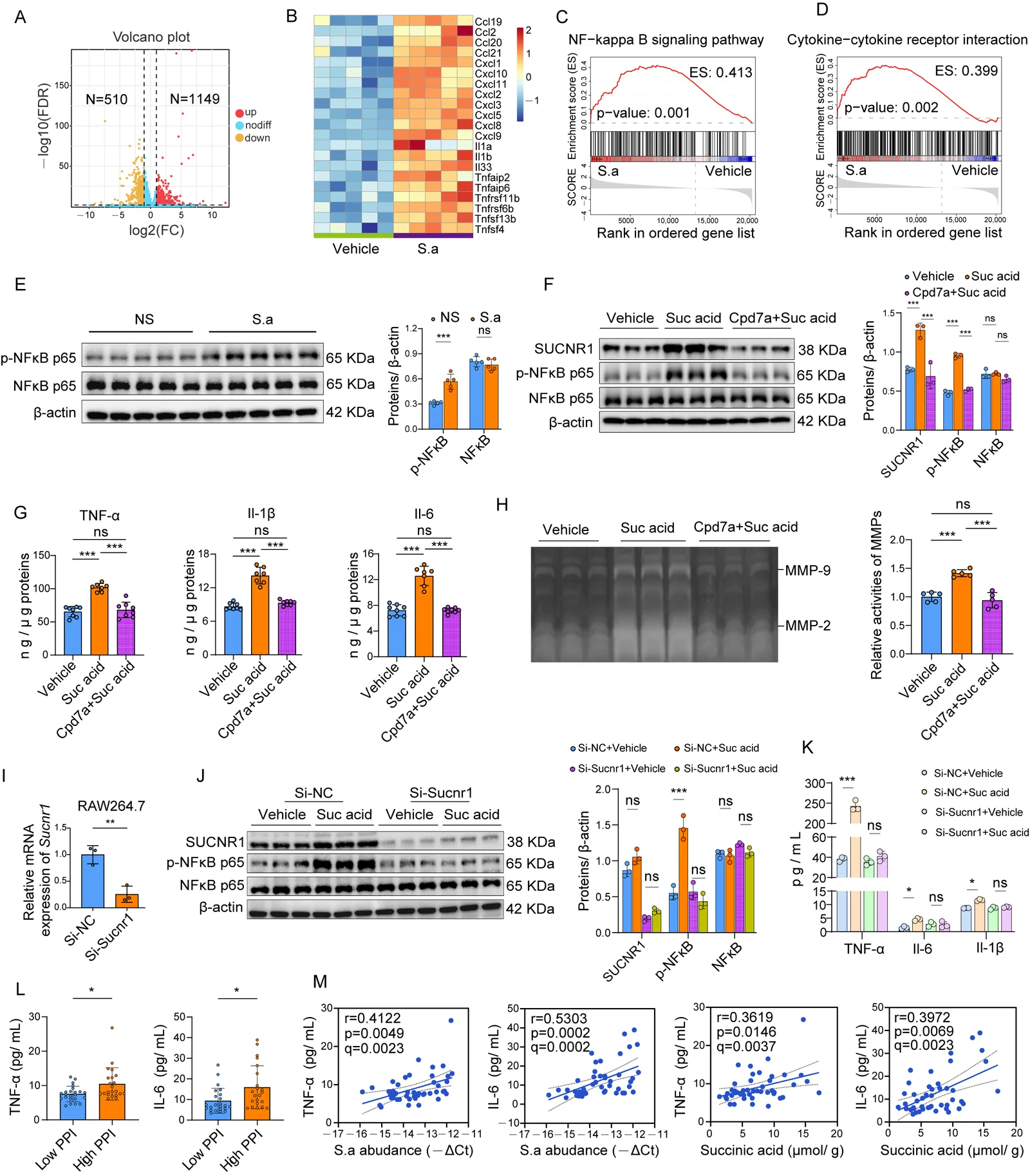
Succinic acid–SUCNR1 signaling activates NF-κB and promotes inflammation to impair anastomotic healing. (**A**) Volcano plot of differentially expressed genes in anastomotic tissues from *S. anginosus* colonized mice versus vehicle controls (*n* = 5). (**B**) Heatmap showing upregulated pro-inflammatory cytokine genes in the *S. anginosus* group. (**C**,**D**) Gene set enrichment analysis (GSEA) revealing significant enrichment of the NF-κB signaling pathway (**C**) and the cytokine-cytokine receptor interaction pathway (**D**) in the *S. anginosus* group. (**E**) Representative Western blots and quantification of phosphorylated NF-κB p65 (p-p65) and total p65 in anastomotic tissues from *S. anginosus*-colonized versus control mice (*n* = 5). (**F**,**G**) Effects of succinic acid treatment with or without SUCNR1 antagonist (Compound **7a**) on: (**F**) SUCNR1/p-p65/p65 levels by Western blot; (**G**) pro-inflammatory cytokine levels (IL-1β, IL-6, TNF-α) by ELISA (*n* = 7–8). (**H**) Gelatin zymography showing MMP-2 and MMP-9 activities in anastomotic tissues from vehicle, succinic acid, and succinic acid + SUCNR1 antagonist groups, with quantification of MMP activity (*n* = 5). (**I**–**K**) In vitro siRNA-mediated knockdown of SUCNR1 in RAW264.7 macrophages: (**I**) qPCR validation of SUCNR1 knockdown efficiency in si-Sucnr1 cells (*n* = 3 independent experiments); (**J**) Western blot validation of SUCNR1 knockdown, p-p65/p65 levels, and β-actin loading control in siNC and si-Sucnr1 cells following succinic acid stimulation, with densitometric quantification (*n* = 3 independent experiments); (**K**) ELISA quantification of pro-inflammatory cytokine secretion in culture supernatant (*n* = 3 independent experiments). (**L**) Serum levels of TNF-α and IL-6 in patients receiving high-dose PPI (DpD ≥ 30 mg/day) versus low-dose PPI (DpD < 30 mg/day) at one week after surgery (*n* = 22 and 23, respectively). (**M**) Spearman correlation analysis of faecal succinic acid levels and *S. anginosus* abundance versus serum TNF-α and IL-6 levels in the same patient cohort (*n* = 45). Spearman correlation coefficients (r) and *p*-values are shown. Data are presented as mean ± SD for bar charts (**E**–**L**). (**A**) Differential expression was analyzed using DESeq2 **(**|log2FC| ≥ 1, FDR < 0.05). (**C**,**D**) GSEA results are shown with enrichment scores (ES) and nominal *p* values. For two-group comparisons (**E**,**I**–**L**), statistical significance was determined by two-tailed Student’s *t*-test. For three-group comparisons (**F**–**H**), statistical significance was determined by one-way ANOVA with Tukey’s post hoc test. (**M**) Spearman correlation coefficients (r), nominal *p* values, and Benjamini–Hochberg FDR-adjusted q values are reported. * *p* < 0.05, ** *p* < 0.01, *** *p* < 0.001; ns, not significant.

To elucidate the molecular mechanisms underlying this impairment, we performed transcriptomic analysis of anastomotic tissues from mice colonized with live *S. anginosus* versus vehicle controls. RNA sequencing identified 1149 upregulated and 510 downregulated genes upon *S. anginosus* colonization (Figure 7A). Notably, key pro-inflammatory cytokine genes were markedly upregulated in the *S. anginosus* group (Figure 7B), and Gene Set Enrichment Analysis (GSEA) revealed significant enrichment of the NF-κB signaling pathway and the cytokine-cytokine receptor interaction pathway (Figure 7C,D). Western blot analysis further confirmed that *S. anginosus* colonization markedly increased phosphorylated NF-κB p65 levels in anastomotic tissues (Figure 7E). Meanwhile, we examined anastomotic tissues from succinic acid-gavaged mice. Consistent with the effects of *S. anginosus* colonization, succinic acid treatment upregulated SUCNR1 expression, markedly increased phosphorylated NF-κB p65 levels (Figure 7F), and elevated the pro-inflammatory cytokines IL-1β, IL-6, and TNF-α in anastomotic tissues (Figure 7G). Furthermore, gelatin zymography revealed that succinic acid treatment enhanced the collagenolytic activity of MMP9 and MMP2 in anastomotic tissues, and this effect was abrogated by SUCNR1 inhibition (Figure 7H). Consistent with these findings, coadministration of the SUCNR1 antagonist also reversed succinic acid-induced NF-κB activation and pro-inflammatory cytokine elevation (Figure 7F,G), supporting the involvement of SUCNR1 in these responses. Because macrophages are known to contribute to the inflammatory phase of anastomotic healing [4], we tested whether macrophages are capable of responding to succinic acid through SUCNR1 in vitro. We performed siRNA-mediated knockdown of SUCNR1 in RAW264.7 macrophages, with knockdown efficiency confirmed by qPCR (Figure 7I), followed by succinic acid stimulation. In control cells (siNC), succinic acid activated NF-κB, as indicated by increased phosphorylated NF-κB p65 levels by Western blots (Figure 7J) and elevated pro-inflammatory cytokines (TNF-α, IL-1β, IL-6) in the culture supernatant (Figure 7K). In contrast, in si-Sucnr1 cells, the succinic acid-induced p-NF-κB p65 level was reduced by approximately 70.20% compared with siNC cells, accompanied by substantial reductions in the secretion of TNF-α, IL-1β, and IL-6 by approximately 83.08%, 44.23%, and 22.20%, respectively (Figure 7J,K). These in vitro data indicate that succinic acid-SUCNR1 signaling can activate NF-κB and drive a pro-inflammatory cytokine response in macrophages.

Finally, to explore whether our murine findings might be relevant in humans. We performed an exploratory clinical association analysis. Patients receiving high-dose PPI exhibited significantly elevated serum TNF-α (mean ± SD: 10.48 ± 4.72 pg/mL vs. 7.55 ± 2.24 pg/mL, *p* = 0.0105) and IL-6 (15.92 ± 10.43 pg/mL vs. 9.40 ± 6.06 pg/mL, *p* = 0.0135) levels at one week after surgery compared with those on low-dose PPI (Figure 7L). Notably, faecal succinic acid and *S. anginosus* levels were both positively correlated with postoperative serum TNF-α and IL-6 levels (Figure 7M). These exploratory clinical findings raise the possibility that the *S. anginosus*-succinic acid axis may be relevant in patients.

## 3. Discussion

In this study, we provide evidence consistent with proton pump inhibitors (PPIs) compromising colorectal anastomotic healing through a previously unrecognised mechanism: gastric acid suppression promotes oral-to-gut enrichment of *S. anginosus*, which secretes succinic acid that activates the SUCNR1-NF-κB signaling in the anastomotic tissue, eliciting a pro-inflammatory response and upregulating MMPs that impair collagen deposition and tissue repair. Our findings provide functional evidence supporting a causal link between a widely overused drug class and a major surgical complication, and delineate a pathogenic axis from pharmacological acid suppression to microbial dysbiosis, immune activation, extracellular matrix degradation, and impaired wound healing.

PPIs profoundly affect the gut microbiome [18,23,24]. A critical question in this field is whether these microbial alterations arise from direct effects of PPIs on gastrointestinal bacteria or from the physiological consequence of gastric acid suppression. Our study provides evidence relevant to this question by using intraperitoneal (i.p.) PPI administration, which bypasses the gastrointestinal lumen while achieving comparable gastric acid suppression. Both oral and i.p. PPI similarly induced oral and gut *S. anginosus* enrichment and impaired anastomotic healing; importantly, these effects were prevented by prior oral microbiota depletion, consistent with a model in which gastric acid suppression promotes oral-to-gut microbial enrichment. Furthermore, PPI still effectively elevated gastric pH in ABX-treated mice (gastric pH remained elevated at 5.31 ± 0.58 in ABX+PPI mice vs. 3.42 ± 0.49 in controls, *p* < 0.0001; Figure 3H), ruling out the possibility that the protective effect of ABX was due to impaired PPI absorption. This supports that the gut microbiota is the essential mediator downstream of PPI-induced acid suppression. To further exclude the confounding effect of oral ABX swabbing on the baseline gut microbiota, 16S re-analysis revealed minor shifts in a few bacterial genera (e.g., *Faecalibacterium* and *Parabacteroides;* Figure S4A), while specific qPCR confirmed that the baseline faecal abundance of *S. anginosus* remained unchanged before and after the oral swabbing (Figure S4B). Although subtle antibiotic carryover cannot be fully excluded, the protective effect of oral ABX swabbing is likely attributable to depleting oral pathogens rather than altering the gut baseline. Consistently, the observation that oral microbiota depletion prevented the phenotype even in i.p. PPI-treated mice indicates that oral bacteria are required, and that acid suppression acts as a sufficient upstream mediator in this model. This aligns with bioreactor evidence showing that PPIs increase *Clostridioides difficile* infection risk primarily by altering pH rather than through direct effects on the gut microbiome [25]. Normally, gastric acid acts as a primary defensive barrier, rapidly killing most orally ingested bacteria [26,27]. When this barrier is compromised by hypochlorhydria or achlorhydria, acid-tolerant or adaptable viable oral bacteria can survive gastric transit and colonize the lower gastrointestinal tract [24,28]. Our findings extend this concept by showing that gastric acid suppression, independent of direct drug exposure, is sufficient to promote the oral-to-gut enrichment of a specific oral pathogen (*S. anginosus*), which subsequently impairs anastomotic healing.

The pathogenic potential of *S. anginosus* has gained increasing recognition beyond its traditional role as an oral commensal. It has been associated with purulent infections and life-threatening bacteremia [29,30,31], and in the gastrointestinal tract, it has emerged as a non-*Helicobacter pylori* pathogen that promotes gastric inflammation and tumorigenesis. Mechanistically, *S. anginosus* leverages its surface protein TMPC to engage with the annexin A2 (ANXA2) receptor on host epithelial cells, activating the MAPK signaling pathway and upregulating proinflammatory chemokines Ccl20 and Ccl8 [32]; its extracellular vesicles (EVs) carrying TMPC and FBP62 activate macrophage polarization and induce acute gastritis [33]. Beyond the stomach, metagenomic and systematic review evidence have implicated *S. anginosus* in colorectal carcinogenesis, with the bacterium enriched in both saliva and faeces of CRC patients and capable of spreading from tumor sites to adjacent normal tissues [34]. In hepatocellular carcinoma, it is also enriched in the gut microbiome of HCC patients and promotes HCC cell migration and tumor progression in orthotopic mouse models by inducing an immunosuppressive microenvironment [35,36].

Our study provides evidence that succinic acid, secreted by *S. anginosus*, may function as a previously underappreciated virulence factor. Succinic acid has emerged as a signaling molecule that activates the G protein-coupled receptor SUCNR1 (also known as GPR91), which is expressed in macrophages, dendritic cells, and epithelial cells [37,38] and has been shown to modulate immune cell recruitment and activation in tissue repair [38]. Our data identify succinic acid as a key candidate effector that is sufficient to contribute to *S. anginosus*-induced anastomotic impairment; heat-inactivated bacteria failed to impair healing, whereas both bacterial supernatant and purified succinic acid recapitulated the defect, and pharmacological SUCNR1 blockade attenuated these effects. However, our data do not establish that bacterially produced succinic acid is necessary for the *S. anginosus* phenotype. Genetic or metabolic impairment of bacterial succinic acid production followed by succinic acid add-back rescue would be required to establish necessity. Mechanistically, succinic acid-SUCNR1 signaling activates NF-κB and upregulates activities of the MMPs, leading to degradation of the collagen-rich extracellular matrix essential for anastomotic integrity. MMPs, particularly MMP-9, have been directly implicated in anastomotic leakage through their ability to degrade type IV collagen and other matrix components [6]. Thus, our findings support a mechanistic link between bacterial metabolism and postoperative complications, and position the succinic acid-SUCNR1 pathway as a potential therapeutic node for mitigating PPI-associated anastomotic complications.

A limitation of our mechanistic conclusion is that macrophages, although capable of responding to succinic acid via SUCNR1 in vitro, are presented only as a plausible cellular mediator rather than a demonstrated in vivo effector cell; the identity of the principal SUCNR1-expressing cell type in anastomotic tissue remains to be determined. Future studies using tissue localization, flow cytometry/single-cell analysis, macrophage depletion, or cell-specific genetic manipulation will be needed to establish their in vivo contribution. In addition, in vivo SUCNR1 dependence was assessed primarily by pharmacological antagonism with Compound **7a**, which may have off-target effects; genetic validation using Sucnr1-deficient mice or a conditional model will be required.

Clinical evidence linking PPI use to anastomotic complications has been reported in esophageal surgery, where preoperative PPI therapy was associated with a 2.5-fold increased risk of anastomotic leaks [39]. While this finding originates from esophageal anastomoses, the underlying mechanism may share a common root: PPI-induced oral dysbiosis enriches pathogenic streptococci, which—whether acting locally in the esophagus after swallowing or after translocation to the colorectum—compromise anastomotic integrity through inflammation and extracellular matrix degradation. This convergence suggests that PPI-associated anastomotic healing risk may extend beyond esophageal surgery. Our exploratory clinical data provide preliminary evidence that high-dose PPI users exhibit faecal enrichment of *S. anginosus* and succinic acid, which correlate with postoperative systemic inflammation. However, because this was an observational, non-randomized cohort of 45 patients, and because the outcomes presented are faecal microbial/metabolic features and postoperative inflammatory markers rather than direct anastomotic healing or leakage, these findings cannot establish the causal chain or clinical anastomotic failure. Nevertheless, these observational findings mirror the signatures observed in our murine model and support further clinical investigation. Together with recent evidence linking higher perioperative PPI daily dose to worse postoperative disease-free survival in colorectal cancer patients [40], these findings support more cautious PPI use in surgical settings.

From a perioperative management perspective, these findings have several implications. First, they provide a mechanistic rationale for restricting PPI use to patients with clear indications (e.g., high risk of stress ulcer bleeding) rather than using them liberally. Second, they suggest that oral microbiota may influence colorectal surgical outcomes. Clinical evidence supports this concern: periodontal disease, which enriches oral *S. anginosus* [41,42], is an independent risk factor for postoperative infectious complications after gastrointestinal cancer surgery (OR = 2.09) [43], and perioperative oral care has been shown to reduce surgical site infection in colorectal cancer patients [44]. Thus, integrating oral health assessment into enhanced recovery protocols represents a logical extension of our findings. Finally, the identification of the succinic acid-SUCNR1-NF-κB signaling reveals multiple potential therapeutic nodes—succinic acid receptor antagonists, NF-κB inhibitors, and oral microbiota decontamination—that could be explored as adjunctive strategies to reduce anastomotic complications.

Several limitations warrant consideration. First, our experiments were conducted in healthy male mice without comorbidities, which may not fully reflect the clinical complexity of colorectal surgery patients. Comorbidities such as diabetes and obesity are highly prevalent and impair baseline anastomotic healing [45,46]; they are also associated with gut microbiota dysbiosis, increased intestinal permeability, and chronic low-grade inflammation [47], which could potentially aggravate PPI-induced microbial dysbiosis. Additionally, circulating succinic acid levels are elevated in obesity and diabetes [48,49], and SUCNR1 expression is increased in macrophages under these conditions [48], potentially sensitizing the SUCNR1 pathway. Furthermore, male sex is an independent risk factor for anastomotic leakage [50], and male rats exhibit less favorable collagen metabolism during early colonic anastomotic healing [51]. Mechanistically, estrogen inhibits NF-κB and promotes M2 macrophage polarization, whereas androgens enhance pro-inflammatory signaling [52,53]; sex hormones also shape gut microbiota composition [54]. Thus, our findings may partly reflect a sex-specific susceptibility, and future studies using comorbid models (e.g., db/db or high-fat diet mice) and both sexes are needed.

Second, the relatively small sample size of our clinical cohort limits the generalizability of the clinical observations, warranting future prospective, multicenter studies with larger cohorts for further validation. Moreover, the 1:1 milligram conversion for PPI classification is a limitation due to the lack of validated conversion formulas for intravenous PPIs; future studies with detailed prescription data are required. Third, other oral pathogenic bacteria—including *Parvimonas micra*, *Fusobacterium nucleatum*, and *Peptostreptococcus* species—have been associated with aggressive gastrointestinal diseases [55,56,57], suggesting their potential contribution to PPI-induced dysbiosis and anastomotic impairment, and their possible synergistic interactions with *S. anginosus* merit further investigation. Finally, although oral antibiotic depletion, FMT, and i.p. PPI experiments collectively support an oral source of the gut *S. anginosus* enrichment, 16S rRNA sequencing cannot resolve strain-level identity; thus, we cannot definitively exclude independent enrichment in both sites. Whole-genome sequencing/SNP comparison or labeled-strain tracking will be required to establish directionality.

## 4. Materials and Methods

### 4.1. Clinical Samples and PPI Dosage Classification

Clinical faecal samples were collected one week after surgery from patients who underwent colorectal resection at Xinqiao Hospital between September 2025 and March 2026, and samples were stored at −80 °C until analysis. Inclusion criteria were (1) age ≥18 years; (2) histologically confirmed colorectal adenocarcinoma; (3) underwent curative-intent resection with primary anastomosis; (4) received perioperative PPI therapy. Exclusion criteria were (1) history of inflammatory bowel disease; (2) preoperative radiotherapy or chemotherapy within 3 months; (3) emergency surgery; (4) American Society of Anesthesiologists (ASA) class ≥ IV. Patients were classified into two groups based on daily PPI dosage (DpD): low-PPI group (DpD < 30 mg/day) and high-PPI group (DpD ≥ 30 mg/day). The PPI dosage classification was based on conventional WHO-defined daily doses (e.g., 30 mg/day for esomeprazole and lansoprazole). The cumulative dose (CD) was defined as the total amount of all PPIs administered from the preoperative preparation period to hospital discharge. When multiple PPI types were recorded, doses were converted on a 1:1 basis. The DpD was then calculated as CD divided by length of hospital stay (LOHS), following the methodology described in a previous study [40]. Notably, CD and DpD based on these algorithms should both be regarded as exploratory variables rather than formally validated clinical indices. The clinical information for enrolled patients was collected from the institutional electronic pathology database and listed in Tables S1 and S2.

### 4.2. Mouse Model of Colonic Anastomosis

Male C57BL/6J mice (8–10 weeks old) were obtained from Slike Jingda Laboratory (Changsha, Hunan, China) and housed under specific pathogen-free conditions at the Xinqiao Hospital Medical Animal Center (12 h light/dark cycle, 21 ± 1 °C, 45 ± 5% humidity). Anaesthesia was induced with 2–3% isoflurane. After abdominal hair removal and disinfection with 10% povidone-iodine, a 2 cm midline laparotomy was performed. The distal colon was partially transected (approximately 90% of the circumference) 1.5 cm proximal to the anus and then closed with interrupted 8-0 polypropylene sutures. Anastomotic integrity was verified by transrectal instillation of warm sterile saline. The abdomen was closed in two layers.

### 4.3. Drug Administration

Esomeprazole magnesium (Cat# S26616, YUANYE, Shanghai, China) was dissolved in 0.9% sterile normal saline (NS, Cat# H20033146, Tiansheng, Chongqing, China). Mice received daily oral gavage of esomeprazole (20 mg/kg, 200 μL) or vehicle (saline) for 10 consecutive days before surgery, and treatment continued daily until sacrifice on postoperative day 6. The 10-day pre-operative regimen was selected to ensure sustained gastric acid suppression and to allow PPI-induced oral-to-gut microbial changes to reach a stable state before anastomosis, consistent with previous PPI-microbiome studies [21,58] and the pharmacology of esomeprazole [15,16]. The 20 mg/kg/day dose was chosen because it is a commonly used mouse dose of PPI that reliably raises gastric pH [59]; effective acid suppression in our model was confirmed by direct gastric pH measurement (Figure 1J and Figure S1H). For intraperitoneal (i.p.) administration, a separate cohort of mice received i.p. injection of esomeprazole (20 mg/kg/day) or vehicle (saline) following the same schedule. Succinic acid (Cat# 110-15-6, Solarbio, Beijing, China) was dissolved in NS and administered by daily oral gavage at a dose of 100 mg/kg, beginning 10 days prior to anastomosis and continuing until sacrifice. For SUCNR1 inhibition, Compound **7a** (succinic acid/succinic acid receptor antagonist 1, Cat# ISA10570, Solarbio, Beijing, China) was dissolved in a vehicle. Mice in the treatment group received Compound **7a** via daily oral gavage at a dose of 10 mg/kg, starting 3 days prior to succinic acid administration and continuing daily until the end of the experiment.

### 4.4. Measurement of Gastric pH

At sacrifice, mice were humanely euthanized, and the whole stomach was rapidly excised and opened along the greater curvature using sterile surgical scissors. Then, as previously described [58], gastric contents were carefully collected into sterile microcentrifuge tubes and resuspended in 500 μL of sterile normal saline. The mixture was fully vortexed and centrifuged at 8000 rpm for 10 min at 4 °C to precipitate insoluble debris. The supernatant was carefully aspirated without sediment contamination, and gastric pH was immediately measured using a calibrated micro-pH electrode (Sanxin, Shanghai, China). The electrode was calibrated with standard buffer solutions (pH 4.0 and 7.0) before use to ensure accuracy.

### 4.5. Anastomotic Healing Score

Anastomotic healing was blindly evaluated by three independent researchers using a standardized 0–4 scoring system based on intra-abdominal findings [3]. A higher score indicates worse healing (0 = well-healed; 4 = anastomotic leak) (Figure 1B).

### 4.6. Biochemical Analyses

Anastomotic tissue homogenates were prepared in two different buffers for the subsequent assays: distilled water for hydroxyproline quantification, and modified RIPA lysis buffer (Cat# P0013B, Beyotime Biotechnology, Shanghai, China) for all other protein analyses. Total protein concentration was determined using a BCA protein assay kit (Cat# BG0020, Baoguang Biotechnology, Chongqing, China). Hydroxyproline content was measured using a commercial colorimetric assay kit (Cat# HBDY96T-9223, Beijing HBDY Biotechnology, Beijing, China) and normalized to tissue wet weight. Fibronectin, TNF-α, IL-6, and IL-1β levels were quantified by enzyme-linked immunosorbent assay (ELISA) using a corresponding kit (Cat# HBDY-0190M1, Cat# HBDY-0132M1, Cat# HBDY-0163M1 and Cat# HBDY-18549M1, Beijing HBDY Biotechnology, Beijing, China) separately and normalized to total protein concentration.

### 4.7. Masson’s Trichrome Staining

For histological analysis, five mice per group were randomly selected from the total surviving animals using a random number generator prior to tissue processing, and this selection was performed by an investigator blinded to the group allocation. Distal colorectal anastomotic tissues were then fixed in 4% paraformaldehyde, paraffin-embedded, and sectioned at 4 μm thickness using a rotary microtome (Leica Microsystems, Wetzlar, Germany). Masson’s trichrome staining was performed according to a standard protocol [60]. Stained sections were examined under a light microscope (Olympus Corporation, Tokyo, Japan) by a board-certified pathologist who was blinded to the experimental groups. Collagen volume fraction was quantified using ImageJ software (Version 1.54f, National Institutes of Health, Bethesda, MD, USA).

### 4.8. Spleen Bacterial Load Quantification

Spleens were harvested under aseptic conditions, weighed, and the spleen-to-body weight ratio was calculated. Each spleen was homogenized in 1 mL of sterile saline. A 200 μL aliquot of homogenate was plated onto blood agar plates (Cat# P0901, Khellin, Jiangmen, China). Plates were incubated aerobically at 37 °C for 48 h, and bacterial loads were quantified by counting colony-forming units (CFU).

### 4.9. Gut Microbiota Depletion

A broad-spectrum antibiotic cocktail (ABX) containing ampicillin (200 mg/kg), vancomycin (100 mg/kg), neomycin (200 mg/kg) and metronidazole (200 mg/kg) was administered by oral gavage (200 μL, once daily) for 5 consecutive days.

### 4.10. Oral Microbiota Depletion

Mice received oral swabbing with ABX treatment twice daily for 3 consecutive days using sterile cotton swabs soaked in 0.5% sodium carboxymethylcellulose (Cat#C14178, psaitong) solution containing ampicillin (20 mg/mL), vancomycin (10 mg/mL), neomycin (20 mg/mL) and metronidazole (20 mg/mL).

### 4.11. Faecal Microbiota Transplantation (FMT)

Recipient mice were treated with a broad-spectrum antibiotic solution by daily oral gavage for 5 days to achieve gut microbiota depletion. Fresh faeces (200 mg) were resuspended in 10 mL sterile 20% glycerol-normal saline, vortexed, settled for 5 min, and the supernatant was centrifuged at 100× *g* for 5 min. The resulting bacterial suspension (200 μL) was administered by daily oral gavage for 10 days before surgery.

### 4.12. 16S rRNA Gene Sequencing

Oral swabs were collected by gently brushing the buccal mucosa and tongue with sterile cytology brushes. Fresh faecal pellets were collected from individual mice. Microbial DNA was extracted using the DNA Extraction Kit (Cat# DNS362-03, Mabio, Guangzhou, China) following the manufacturer’s instructions. The V4 region of the 16S rRNA gene was amplified using specific primers (V4F: 5′-GTGTGYCAGCMGCCGCGGTAA-3′; V4R: 5′-CCGGACTACNVGGGTWTCTAAT-3′). A no-template negative control was included in every PCR run to monitor reagent contamination (no bands were observed). Amplicons were quality-checked by 2% agarose gel electrophoresis, purified with AMPure XP Beads (Beckman Coulter, Brea, CA, USA), and quantified using a Qubit 3.0 Fluorometer (Thermo Fisher Scientific, Waltham, MA, USA). Sequencing libraries were constructed with the Illumina DNA Prep Kit (Illumina, San Diego, CA, USA) and validated using an ABI StepOnePlus Real-Time PCR System (Life Technologies, Carlsbad, CA, USA). Paired-end sequencing (PE250) was performed on an Illumina NovaSeq 6000 platform (Illumina, San Diego, CA, USA). The raw sequencing data were analyzed with the QIIME2 platform (v2024.2). ASVs were generated using DADA2 (v1.14.1), and taxonomy was assigned using the SILVA 138.2 database. Samples were rarefied to a uniform depth of 35,000 reads per sample.

### 4.13. Colony Picking and Bacterial Identification

*S. anginosus* strains isolated from mouse oral and faecal samples were used specifically for full-length 16S rRNA sequencing and phylogenetic comparison. Specifically, the isolation and in vitro culture of *S. anginosus* were performed as previously described [61]. Frozen saliva and faecal specimens were thawed in an aerobic chamber and subjected to 10-fold serial dilutions in sterile phosphate-buffered saline (PBS). A volume of 100 μL of each appropriate dilution was spread onto brain-heart infusion (BHI) agar plates and incubated aerobically at 37 °C for 24 to 48 h. Daily colony observation and picking were performed for successive purification. Colonies were selected based on distinct morphological characteristics, including colony size, perimeter, circularity, convexity, color, and consistency. Selected colonies were streaked onto fresh BHI agar plates and incubated at 37 °C in an aerobic chamber for propagation. Purified colonies were sent to Tsingke Biotechnology for full-length 16S rRNA gene sequencing, and sequencing results were subjected to homology alignment.

### 4.14. Quantitative Real-Time PCR (qPCR) Analysis

DNA extracted from oral swabs and faeces was used for qPCR to quantify *Streptococcus anginosus* and total bacteria. The *S. anginosus* primers were designed based on a previous study [32], and their specificity was validated by conventional PCR followed by 2% agarose gel electrophoresis. Using *S. anginosus*, *S. parasanguinis*, *S. intermedius*, and a no-template control (NTC), only *S. anginosus* yielded a specific band (Figure S4C). The primers used were as follows:

16S rRNA primers: Forward 5′-GTGSTGCAYGGYTGTCGTCA-3′; Reverse 5′-ACGTCRTCCMCACCTTCCTC-3′.

*Streptococcus anginosus* primers: Forward 5′-GAACGGGTGAGTAACGCGTAGGTA-3′; Reverse 5′-AAGCATCTAACATGTGTTACATAC-3′.

For SUCNR1 knockdown efficiency in RAW264.7 macrophages, qPCR was performed using mouse Sucnr1 primers: Forward 5′-TGGTTGGCAACAGAGGCTAT-3′; Reverse 5′-TGACATTCCCAAGCAGTCCA-3′, with β-actin as an internal control: Forward 5′-CATCCACGAAACTACCTTCAACTC-3′; Reverse 5′-GAGCCGCCGATCCACACG-3′. Results were normalized to total bacterial 16S rDNA or endogenous β-actin and expressed as relative abundance using the 2^−ΔΔCt^ method.

### 4.15. Streptococcus anginosus Culture and Colonization

The *Streptococcus anginosus* strain ATCC 33397 was obtained from BeNa Culture Collection (BNCC; Cat# BNCC335670) and used for all functional experiments (live bacteria administration, heat inactivation, and supernatant preparation). The bacteria was cultured in brain heart infusion (BHI) broth at 37 °C under aerobic conditions to the mid-log phase [32]. Bacteria were harvested, washed twice with sterile PBS, and adjusted to 2 × 10^9^ CFU/mL. For colonization, mice received 200 μL of bacterial suspension by oral gavage once daily for 10 consecutive days before anastomosis. For heat-inactivated bacterial experiments, the suspension was heated at 70 °C for 30 min. For supernatant administration, bacterial supernatant was collected by centrifugation (5000× *g*, 15 min) and filtered through a 0.22 μm filter.

### 4.16. Metabolomic Profiling

Non-targeted metabolomic analysis was conducted by Nomagic Metabolomics Inc, China. Mouse faeces were homogenized in 80% methanol and centrifuged. Supernatants were subjected to LC-MS/MS analysis using a UHPLC system coupled with a Q-Exactive Orbitrap mass spectrometer. Pooled quality-control (QC) samples, prepared by mixing equal aliquots of all samples, were injected at regular intervals throughout the analytical batch; blank samples were injected to monitor carry-over, and the injection order was randomized. Features were retained only if consistently detected in pooled QC samples, and QC reproducibility was monitored before data analysis. Raw data were processed using Proteowizard and XCMS, and metabolites were identified by matching accurate mass and MS/MS spectra against public databases (HMDB, MassBank, LipidMaps, mzCloud, and KEGG).

### 4.17. Transcriptome Sequencing and Analysis

Total RNA was extracted from anastomotic tissues using TRIzol reagent (Thermo Scientific, Waltham, MA, USA). Purified total RNA was used to construct libraries and sequenced on an Illumina NovaSeq 6000 platform (Novogene Co., Ltd., Beijing, China). Differential expression analysis was performed with DESeq2 (|log2FC| ≥ 1, FDR < 0.05).

### 4.18. Succinic Acid Quantification

Succinic acid levels in faecal samples and bacterial culture supernatants were measured using a commercial colorimetric assay kit (Cat# KQ142774, Keqiao Biotech, Suzhou, China). Faecal samples were homogenized in deionized water (1:5, *w*/*v*) and centrifuged at 3000× *g* for 10 min; bacterial supernatant was centrifuged directly under the same conditions. The resulting supernatants were assayed according to the manufacturer’s instructions.

### 4.19. Cell Experiments

Murine macrophage cell line RAW264.7 (Cat# CL-0190; Procell, Wuhan, China) were cultured in DMEM medium (Cat# E600003, Sangon Biotech, Shanghai, China) supplemented with 10% fetal bovine serum (Cat# E600051, Sangon Biotech, Shanghai, China) and 1% penicillin-streptomycin (Cat# E607011, Sangon Biotech, Shanghai, China) at 37 °C in a 5% CO_2_ incubator. For siRNA-mediated knockdown, cells were seeded in 6-well plates at 5 × 10^5^ cells/well and transfected with 50 nM of si-Sucnr1 (sense: 5-AGAGAAGGACCUAGAGAUUTT-3, antisense: 5-AAUCUCUAGGUCCUUCUCUTT-3) or si-NC(sense: 5-UUCUCCGAACGUGUCACGUTT-3, antisense: 5-ACGUGACACGUUCGGAGAATT-3) using riboFECT CP Transfection Kit (Cat# C10511-05, RiboBio, Guangzhou, China) according to the manufacturer‘s protocol. At 48 h post-transfection, cells were treated with 800 μM sodium succinic acid or DMSO vehicle for 36 h. The concentration of 800 μM succinic acid was selected based on published studies using similar concentration ranges in macrophages (0.5–2 mM) [62,63]. Knockdown efficiency was confirmed by qPCR and Western blot. Culture supernatants were collected for cytokine measurement by ELISA.

### 4.20. Gelatin Zymography

Activities of MMPs in anastomotic tissues were assessed by gelatin zymography, which detects proteolytic activity based on gelatin degradation during electrophoresis, allowing simultaneous identification of MMP-2 and MMP-9 by their molecular weights. All procedures were performed using a commercial gelatin zymography assay kit (Cat# P1700, Applygen, Beijing, China) following the manufacturer’s instructions. Band intensities were quantified using ImageJ software.

### 4.21. Western Blot

Anastomotic tissues were lysed in RIPA buffer containing protease and phosphatase inhibitors. Equal amounts of protein were separated by SDS-PAGE, transferred to PVDF membranes (Cat# IPVH00010, Merck Millipore, Darmstadt, Germany), and probed with primary antibodies against SUCNR1 (1:1000, Cat# 347445, Zenbio, Chengdu, China), phospho-NF-κB p65 (1:1000, Cat# 3039, CST, Danvers, MA, USA), NF-κB p65 (1:1000, Cat# 8242, CST, Danvers, MA, USA), and β-actin (1:1000, Cat# R380624, Zenbio, Chengdu, China). Signals were visualized using an ECL reagent (Cat# BL520B, bioSharp, Hefei, China) and imaged with an Azure c300 chemiluminescence system. Quantification was performed using ImageJ (Version 1.54f, National Institutes of Health, Bethesda, MD, USA).

### 4.22. Statistical Analysis

Statistical analyses were performed using GraphPad Prism v10.4.1 (GraphPad Software, La Jolla, CA, USA) and R v4.4.2 (R Foundation for Statistical Computing, Vienna, Austria). Normality (Shapiro–Wilk test) and variance homogeneity (Levene’s test) were assessed prior to parametric testing. For continuous variables meeting these assumptions, two-tailed unpaired Student’s t-tests were used for two-group comparisons, and one-way ANOVA with Tukey’s post hoc test for comparisons among three or more groups. When assumptions were violated, non-parametric alternatives were used (Mann–Whitney U test or Kruskal–Wallis test with Dunn’s post hoc test). For multiplicity, the primary animal experiments were pre-specified confirmatory tests of a single hypothesis; thus, no global correction was applied. For exploratory correlation analyses (Figure 7M), Benjamini–Hochberg FDR correction was applied, reporting both nominal *p* and adjusted q values. *p*-values are indicated as * *p* < 0.05, ** *p* < 0.01, and *** *p* < 0.001; ns, not significant.

### 4.23. Animal Flow and Exclusions

For all experiments, the number of animals allocated to each group was predefined. In the colorectal anastomosis model, mice that developed severe anastomotic leakage (healing score 4) and died before the planned postoperative day 6 harvest were recorded. These mice were included in the macroscopic anastomotic healing score analysis (scored as 4), but were necessarily excluded from tissue-based analyses (hydroxyproline, fibronectin, Masson staining, and splenic bacterial load) due to the lack of viable tissue samples. No animals were excluded post hoc for any other reason. A detailed per-group animal flow is provided in Table S4.

### 4.24. Randomization and Blinding

Mice were randomized using a computer-generated sequence and matched for age, sex, and body weight. Cages were randomly assigned to treatment groups. The investigator administering treatments was not blinded. However, the surgeon, the investigators performing molecular assays, and the statistician were all blinded to group allocation.

## 5. Conclusions

In summary, this study provides evidence consistent with PPI-induced gastric acid suppression promoting oral-to-gut enrichment of *S. anginosus*, which secretes succinic acid to activate the SUCNR1-NF-κB signaling, leading to impaired collagen deposition and anastomotic healing. These findings suggest caution regarding the overuse of PPIs in surgical patients and support the re-evaluation of current prescribing practices. Furthermore, this study raises the possibility that interventions targeting oral microbiota or succinic acid signaling could be explored as adjunctive strategies to improve anastomotic healing.

## Data Availability

The transcriptome, 16S rDNA, and metabolome data in this study have been deposited in the China National GeneBank Sequence Archive (CNSA; https://db.cngb.org/cnsa/, accessed on 5 November 2026) under accession numbers CNP0009657, CNP0009660, and CNP0009789, respectively. Other data relevant to the study are available from the corresponding authors upon reasonable request.

## Supplementary Materials

The following supporting information can be downloaded at: https://www.mdpi.com/article/10.3390/ijms27219988/s1.
