# Supplementary material for: Proton Pump Inhibitors Promote Oral-to-Gut Enrichment of S. anginosus and Impair Anastomotic Healing via Succinic Acid-SUCNR1-NF-κB Signaling Axis
Source: Int J Mol Sci. 2026 Nov 7;27(21):9988. doi: 10.3390/ijms27219988 (PMC13764325; doi:10.3390/ijms27219988)
Supplement: Supplementary file 1 [file ijms-27-09988-s001.zip › Supplementary_Material_Revised.pdf]

# *Supplementary Material*

## Supplementary Figures

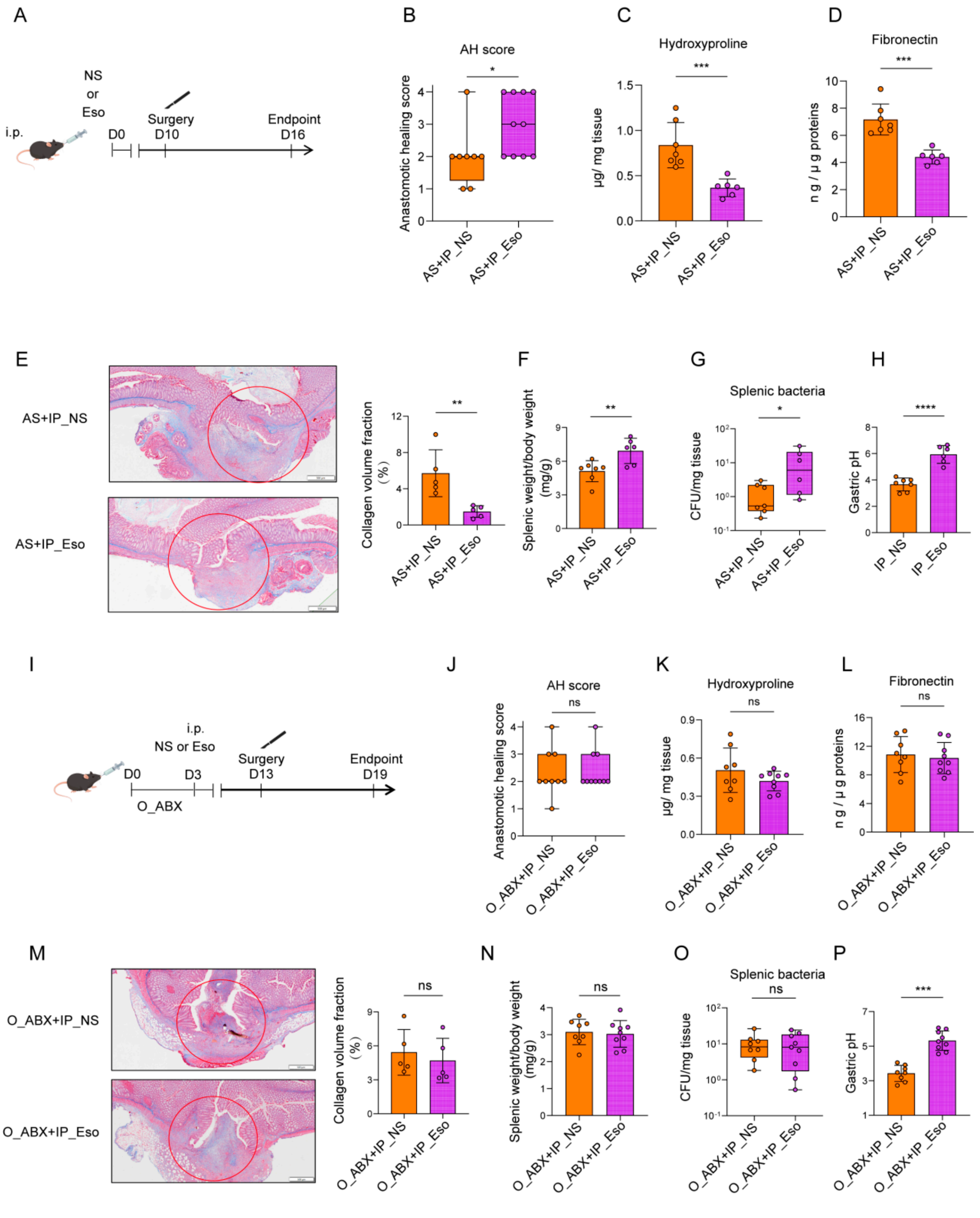

**Figure S1. Intraperitoneal injection with PPI impairs colorectal anastomotic healing and this effect is abrogated by prior depletion of oral microbiota.**

**(A)** Schematic of the experimental protocol for Eso administration via intraperitoneal injection (i.p.): mice were administered NS or Eso via daily i.p. for 10 consecutive days before colorectal anastomosis, and target tissues were collected on postoperative day 6 for subsequent experimental detection. **(B–G)** Healing parameters at the anastomotic site: **(B)** anastomotic healing score (AHS, n = 8–10), **(C)** hydroxyproline content and **(D)** fibronectin levels (n = 6–7). **(E)** Representative Masson's trichrome staining of anastomotic tissues (blue: collagen, red circle: anastomosis site; scale bars: 500 μm) and corresponding collagen volume fraction quantification (n = 5). **(F)** Quantification of the splenic weight-to-body weight ratio (mg/g) in mice with i.p. NS or Eso. **(G)** Quantitative bacterial load in the spleen (n = 6–7). **(H)** Gastric pH (n = 6–7). **(I)** Schematic of the experimental protocol for i.p. PPI administration with ABX oral swabbing: mice were given 3 consecutive days of oral swabbing with ABX, followed by 10-day daily NS or PPI i.p. for 10 consecutive days before colorectal anastomosis, and target tissues were collected on postoperative day 6. **(J–O)** Healing parameters at the anastomotic site: **(J)** anastomotic healing score (AHS, n = 9–10), **(K)** hydroxyproline content and **(L)** fibronectin levels (n = 8–9). **(M)** Representative Masson's trichrome staining of anastomotic tissues (blue: collagen, red circle: anastomosis site; scale bars: 500 μm) and corresponding collagen volume fraction quantification (n = 5). **(N)** Quantification of the splenic weight-to-body weight ratio (mg/g) in mice. **(O)**

Quantitative bacterial load in the spleen (n = 8–9). **(P)** Gastric pH (n = 8–9). Data were presented as mean ± SD for bar chart data (C, D, E, F, H, K, L, M, N, P), or as box-and-whisker plots (min to max, with median line) for AHS (B, J) and splenic bacterial load (G, O). Statistical significance for continuous variables (C–F, H, K–N, P) was determined by the two-tailed Student's t-test, whereas the anastomotic healing score (B, J) and splenic bacterial load (G, O) were analyzed using the Mann–Whitney U test (* $p < 0.05$, ** $p < 0.01$, *** $p < 0.001$; ns, not significant).

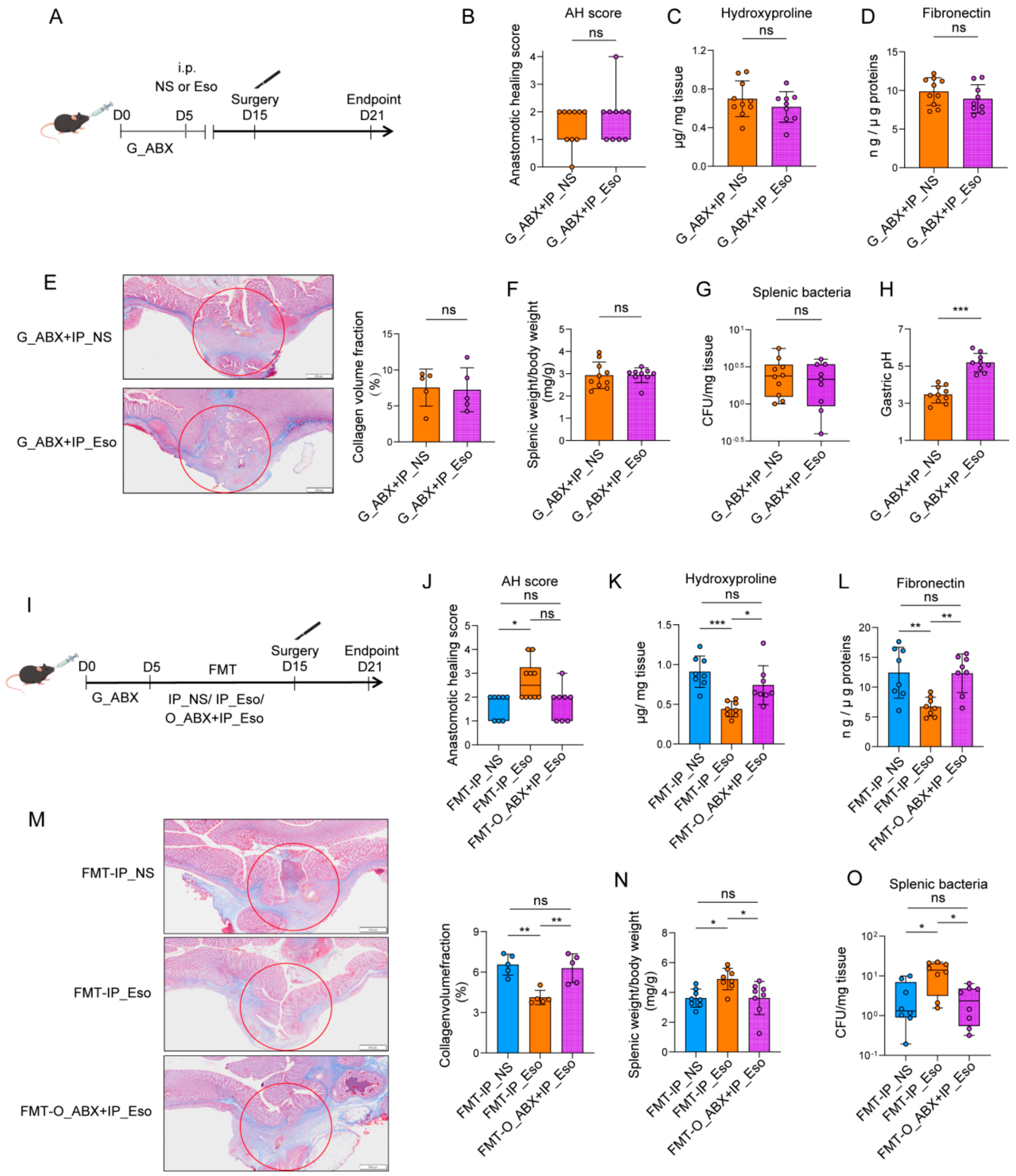

**Figure S2. Intraperitoneal injection with PPI-induced healing impairment depends on gut microbiota and is transmissible via faecal microbiota transplantation.**

**(A)** Schematic of the experimental protocol for i.p. of PPI administration with ABX gavage: mice were given 5 consecutive days of ABX gavage, followed by 10-day daily NS or PPI i.p. for 10 consecutive days before colorectal anastomosis, and target

tissues were collected on postoperative day 6. **(B–G)** Healing parameters at the anastomotic site: **(B)** anastomotic healing score (AHS, n = 10), **(C)** hydroxyproline content and **(D)** fibronectin levels (n = 9–10). **(E)** Representative Masson's trichrome staining of anastomotic tissues (blue: collagen, red circle: anastomosis site; scale bars: 500 μm) and corresponding collagen volume fraction quantification (n = 5). **(F)** Quantification of the splenic weight-to-body weight ratio (mg/g) in mice. **(G)** Quantitative bacterial load in the spleen (n = 9–10). **(H)** Gastric pH (n = 9–10). **(I)** Schematic of the experimental protocol for the mouse donor FMT experiment via i.p.: mice received 5-day pre-treatment with ABX, followed by 10 days of FMT from donor mice treated with NS, i.p. Eso, or oral ABX swabbing combined with i.p. Eso injection. After the 10-day FMT intervention, mice underwent colorectal anastomosis surgery, and target tissues were harvested on postoperative day 6. **(J–O)** Healing parameters at the anastomotic site: **(J)** anastomotic healing score (AHS, n = 8–10), **(K)** hydroxyproline content and **(L)** fibronectin levels (n = 8). **(M)** Representative Masson's trichrome staining of anastomotic tissues (blue: collagen, red circle: anastomosis site; scale bars: 500 μm) and corresponding collagen volume fraction quantification (n = 5). **(N)** Quantification of the splenic weight-to-body weight ratio (mg/g) in mice. **(O)** Quantitative bacterial load in the spleen (n = 8). Data were presented as mean ± SD for bar chart data (C, D, E, F, H, K, L, M, N), or as box-and-whisker plots (min to max, with median line) for AHS (B, J) and splenic bacterial load (G, O). For two-group comparisons (B–H), statistical significance for continuous variables (C–F, H) was determined by the two-tailed Student's t-test,

whereas AHS (B) and splenic bacterial load (G) were analyzed using the Mann–Whitney U test. For three-group comparisons (J–O), continuous variables (K–N) were analyzed using one-way ANOVA with Tukey's post-hoc test, whereas AHS (J) and splenic bacterial load (O) were analyzed using the Kruskal–Wallis test with Dunn's post-hoc test (*p < 0.05, **p < 0.01, ***p < 0.001; ns, not significant).

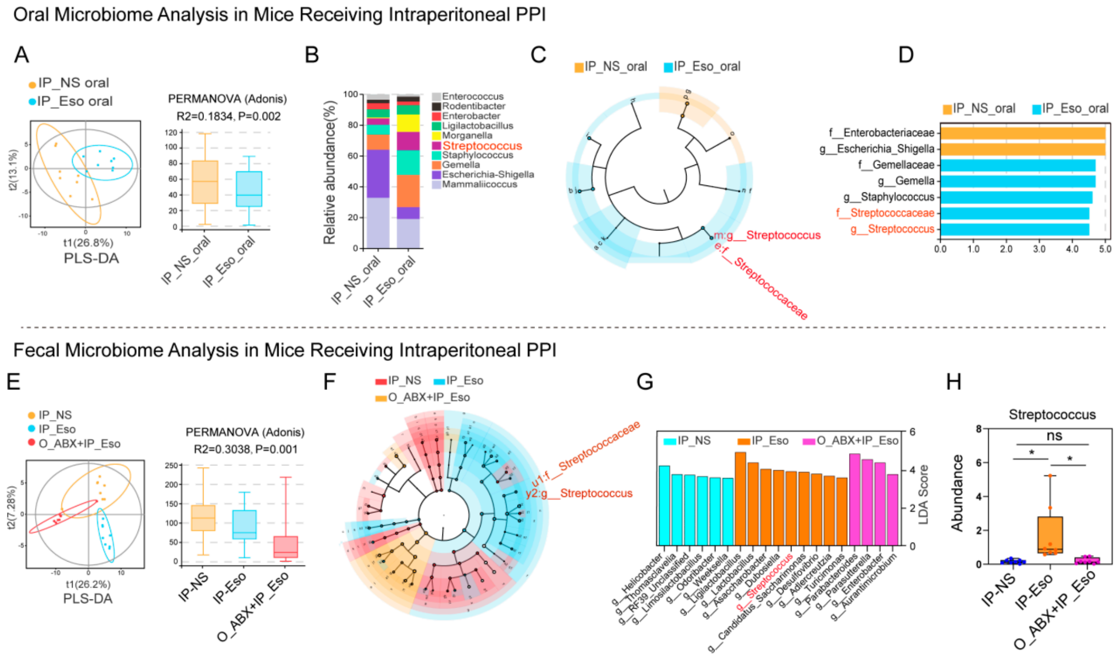


**Figure S3. Intraperitoneal injection with PPI modifies oral and gut microbial communities, characterized by *Streptococcus* enrichment.**

(A) Partial least squares discriminant analysis (PLS-DA) plot demonstrating the clear separation of oral microbiota community structure between the i.p. of normal saline (IP_NS_oral group) and esomeprazole (IP_Eso_oral group) (n = 8). The corresponding PERMANOVA (Adonis) results are shown alongside ($R^2 = 0.1834$, P = 0.002). **(B)** Stacked bar chart showing the relative abundance of dominant bacterial

genera in oral microbiota from each experimental group. **(C)** Cladogram generated from linear discriminant analysis effect size (LEfSe) analysis. **(D)** Linear discriminant analysis (LDA) score distribution plot, histogram shows the taxa with LDA scores greater than 4.0. **(E)** Partial least squares discriminant analysis (PLS-DA) plot demonstrating the distinct separation of gut microbiota community structure among the normal saline intraperitoneal injection (IP_NS group), esomeprazole (proton pump inhibitor, PPI) intraperitoneal injection (IP_Eso group), and oral ABX swabbing combined with PPI intraperitoneal injection (O_ABX+IP_Eso group) (n = 7–8). The corresponding PERMANOVA (Adonis) results are shown alongside ($R^2$ = 0.3038, P = 0.001). **(F)** Cladogram generated from linear discriminant analysis effect size (LEfSe) analysis, displaying the differentially abundant taxonomic clades among groups. **(G)** Histogram of linear discriminant analysis (LDA) scores, histogram shows the taxa with LDA scores greater than 3.0. **(H)** Box plot showing the relative abundance of *Streptococcus* in the gut microbiota of mice from each group.

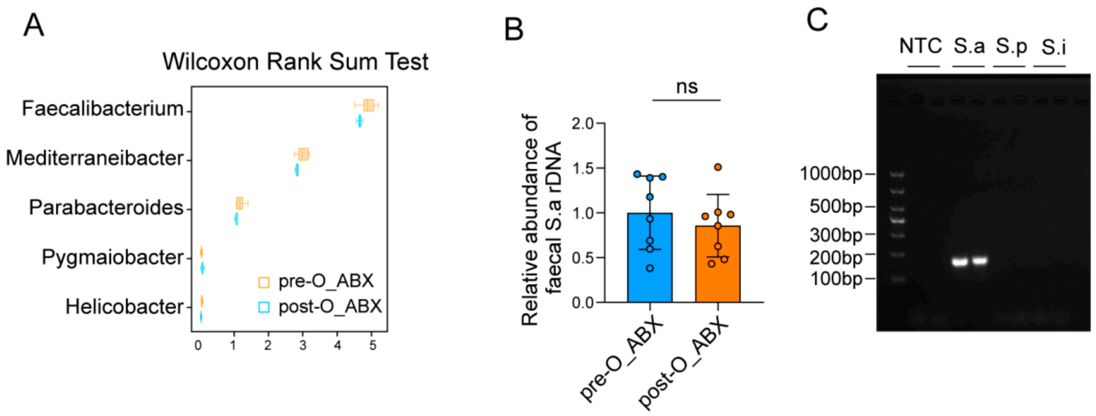


**Figure S4. Effects of oral ABX swabbing on faecal microbiota and validation of *S. anginosus* primer specificity.**

(A) Box plots showing the differential abundance of bacterial genera in faeces before (pre-O_ABX) and after (post-O_ABX) oral ABX swabbing, analyzed using the Wilcoxon rank-sum test. (B) Relative abundance of faecal S. a rDNA in mice before (pre-O_ABX) and after (post-O_ABX) oral ABX swabbing (n = 8). Data are presented as mean ± SD. Statistical significance was determined by the two-tailed Student's t-test (ns, not significant). (C) Agarose gel electrophoresis validating the specificity of the *S. anginosus* (S. a) species-specific primers. Lanes: NTC (no-template control), S. a (*S. anginosus*), S. p (*S. parasanguinis*), and S. i (*S. intermedius*). A specific band of the expected size was only observed in the S. a lane, confirming primer specificity.

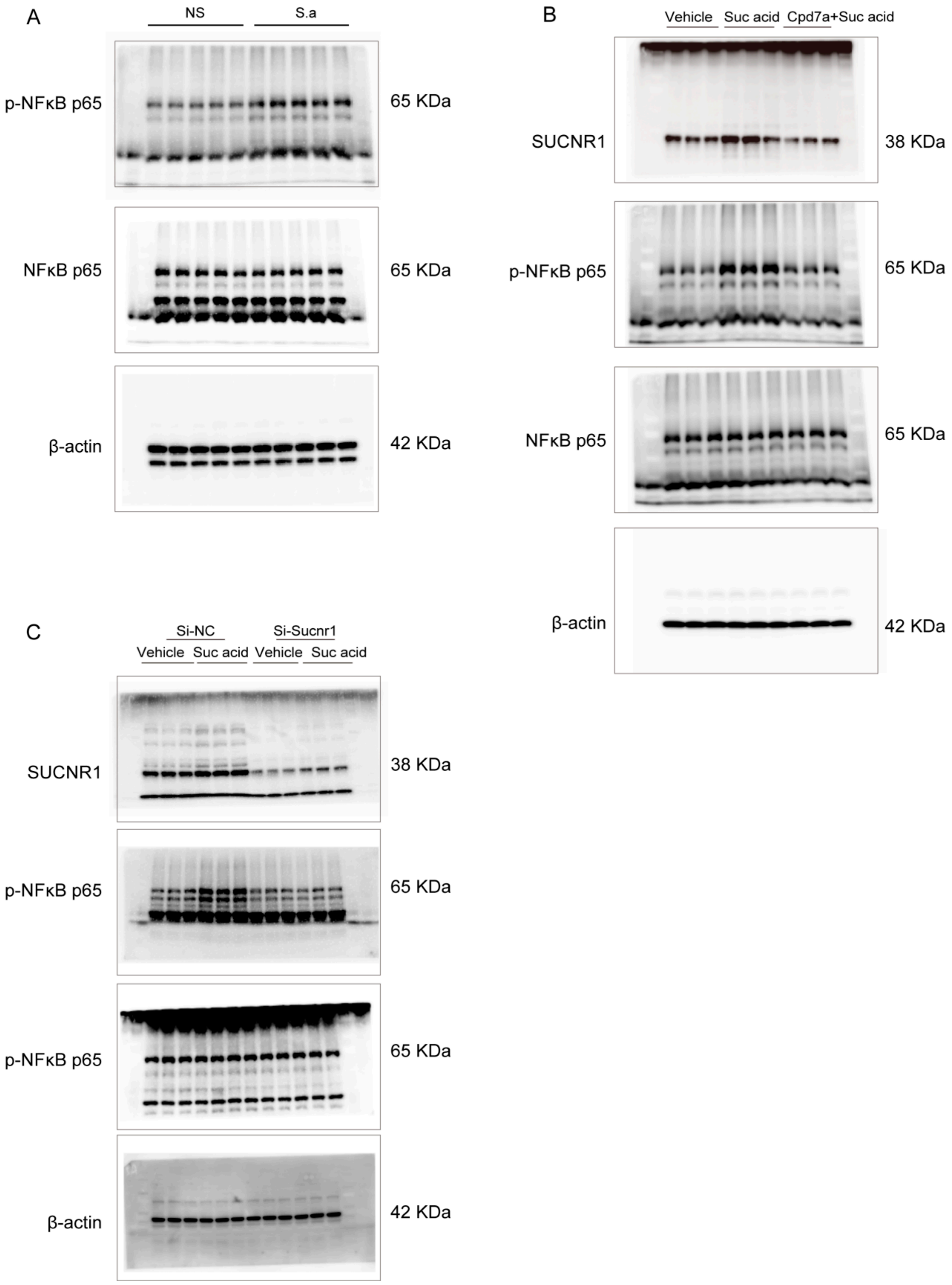


**Figure S5. The full scan of the western blot in Figure 7.**

**Supplementary table**

**Table S1. Clinical characteristics of patients grouped by PPI dosage**

| **Characteristics** | **High-dose PPI (n = 22)** | **Low-dose PPI (n = 23)** | **P value** |
|---|---|---|---|
| PPI cumulative dose (mg), Mean ± SD | 519.09 ± 234.15 | 118.26 ± 64.85 | **<0.001** |
| PPI daily dose (DpD, mg/day), Mean ± SD | 41.14 ± 13.26 | 8.95 ± 4.74 | **<0.001** |
| Length of hospital stay (LOHS, days), Mean ± SD | 12.5 ± 3.61 | 13.52 ± 3.17 | 0.318 |
| Age (years), Mean ± SD | 60.91 ± 11.27 | 59.86 ± 10.61 | 0.751 |
| Gender (M/F) | 15/7 | 13/10 | 0.542 |
| BMI (kg/m²), Mean ± SD | 22.77 ± 3.01 | 23.19 ± 3.00 | 0.639 |
| Diabetes (%) | 2 (9.1%) | 1 (4.3%) | 0.608 |
| Hypertension (%) | 4 (18.2%) | 3 (13.0%) | 0.699 |
| Smoking (%) | 5 (22.7%) | 7 (30.4%) | 0.738 |
| Distance from anal verge (cm), Mean ± SD | 7.10 ± 3.03 | 7.26 ± 3.05 | 0.860 |
| Tumor size (cm), Mean ± SD | 2.98 ± 1.05 | 2.75 ± 1.36 | 0.530 |
| TNM stage (I/II/III) | 1/5/16 | 3/8/12 | 0.339 |
| ASA grade (II/III) | 17/5 | 15/8 | 0.514 |
| Surgical approach (open/laparoscopic) | 0/22 | 0/23 | 1.000 |
| Operation time (min), Mean ± SD | 184.9 ± 78.72 | 199.57 ± 74.37 | 0.501 |
| Blood loss (mL), Mean ± SD | 127.73 ± 77.82 | 157.39 ± 90.61 | 0.246 |

*Data are presented as mean ± SD for continuous variables, or n (%) for categorical variables. P values were determined by Student's t-test (normally distributed continuous variables), Mann-Whitney U test (non-normally distributed continuous

variables), or Fisher's exact test (categorical variables). Statistically significant differences (P < 0.05) are highlighted in bold. DpD, dosage per day; LOHS, length of hospital stay.

**Table S2. Distribution of specific PPI agents in the clinical cohort.**

| PPI Agent (Intravenous) | High-dosePPI (n=22) | Low-dosePPI (n=23) | Total(n=45) |
|---|---|---|---|
| Esomeprazole | 2 | 4 | 6 |
| Omeprazole | 1 | 2 | 3 |
| Pantoprazole | 14 | 14 | 28 |
| Lansoprazole | 5 | 3 | 8 |

All patients received intravenous PPI therapy during the perioperative period for stress ulcer prophylaxis.
